# Defining super-enhancers by highly ranked histone H4 multi-acetylation levels identifies transcription factors associated with glioblastoma stem-like properties

**DOI:** 10.1186/s12864-023-09659-w

**Published:** 2023-09-27

**Authors:** Nando D. Das, Jen-Chien Chang, Chung-Chau Hon, S. Thomas Kelly, Shinsuke Ito, Marina Lizio, Bogumil Kaczkowski, Hisami Watanabe, Keisuke Katsushima, Atsushi Natsume, Haruhiko Koseki, Yutaka Kondo, Aki Minoda, Takashi Umehara

**Affiliations:** 1 Laboratory for Epigenetics Drug Discovery, RIKEN Center for Biosystems Dynamics Research, Yokohama, Japan; 2 Laboratory for Cellular Epigenomics, RIKEN Center for Integrative Medical Sciences (IMS), Yokohama, Japan; 3 Laboratory for Genome Information Analysis, RIKEN IMS, Yokohama, Japan; 4 Laboratory of Developmental Genetics, RIKEN IMS, Yokohama, Japan; 5 Laboratory for Applied Regulatory Genomics Network Analysis, RIKEN IMS, Yokohama, Japan; 6 Division of Cancer Biology, Nagoya University Graduate School of Medicine, Nagoya, Japan; 7 Department of Neurosurgery, Nagoya University Graduate School of Medicine, Nagoya, Japan; 8 Immune Regulation, Advanced Research Departments, Graduate School of Medicine, Chiba University, Chiba, Japan; 9 Department of Cell Biology, Faculty of Science, Radboud Institute for Molecular Life Sciences, Radboud University, Nijmegen, Netherlands; 10 PRESTO, Japan Science and Technology Agency, Kawaguchi, Saitama Japan

**Keywords:** Epigenetics, Histone acetylation, Inhibitor, Nucleosome, Tumorigenesis

## Abstract

**Background:**

Super-enhancers (SEs), which activate genes involved in cell-type specificity, have mainly been defined as genomic regions with top-ranked enrichment(s) of histone H3 with acetylated K27 (H3K27ac) and/or transcription coactivator(s) including a bromodomain and extra-terminal domain (BET) family protein, BRD4. However, BRD4 preferentially binds to multi-acetylated histone H4, typically with acetylated K5 and K8 (H4K5acK8ac), leading us to hypothesize that SEs should be defined by high H4K5acK8ac enrichment at least as well as by that of H3K27ac.

**Results:**

Here, we conducted genome-wide profiling of H4K5acK8ac and H3K27ac, BRD4 binding, and the transcriptome by using a BET inhibitor, JQ1, in three human glial cell lines. When SEs were defined as having the top ranks for H4K5acK8ac or H3K27ac signal, 43% of H4K5acK8ac-ranked SEs were distinct from H3K27ac-ranked SEs in a glioblastoma stem-like cell (GSC) line. CRISPR-Cas9–mediated deletion of the H4K5acK8ac-preferred SEs associated with *MYCN* and *NFIC* decreased the stem-like properties in GSCs.

**Conclusions:**

Collectively, our data highlights H4K5acK8ac’s utility for identifying genes regulating cell-type specificity.

**Supplementary Information:**

The online version contains supplementary material available at 10.1186/s12864-023-09659-w.

## Background

The state of a mammalian cell, such as its stem-like properties, is determined by cell type–specific gene expression, which is controlled through gene regulatory *cis*-elements, such as enhancers and super-enhancers (SEs) [1–4]. Enhancers are *cis*-elements with a wide variety of DNA sequences that are bound by various transcription factors (TFs), and facilitate gene transcription [2, 4, 5]. SEs are clusters of enhancers occupied by exceptionally high densities of transcriptional coactivators and/or active chromatin marks; they usually facilitate a higher level of gene transcription than that from other enhancers [3, 6, 7]. The formation of an SE is often observed at TF-encoding genes in a cell type–specific manner, including at several oncogenes in cancer cells [3, 8, 9]. For example, aberrant expression of c-*MYC*, which encodes a multifunctional TF, is frequently associated with cell type–specific formation of an SE in many cancers [8–10].

Currently, SEs are defined mainly based on high-level enrichment for binding of transcriptional coactivators, *e.g.*, a bromodomain (BRD) and extra-terminal domain (BET) family protein BRD4 and Mediator complex subunit 1 (MED1) and/or the presence of the active chromatin mark of acetylation of histone H3 on lysine 27 (H3K27ac) [3, 6, 7, 11]. BRD4 functions as an epigenetic reader that binds to acetylated lysine residues of chromatic histones [12]. The binding of BRD4 to the acetylated histones at an enhancer or SE, or at a promoter, which is a gene regulatory *cis*-element containing the transcription start site (TSS) [13], recruits RNA polymerase II (RNAP II) to the promoter, facilitating the expression of the associated gene [14, 15]. Notably, altered enrichments of these transcriptional coactivators and/or H3K27ac are often associated with the aberrant formation of SEs at oncogenes in cancer cells, including cancer stem cells [6–10, 16].

BRD4 and other BET family proteins share two tandem BRDs (BD1 and BD2) [17] that primarily bind to histone H4 multi-acetylated at K5, K8, K12, and K16 [18, 19]. In particular, BD1 preferentially binds to histone H4 tails containing more than one acetylation within a span of 1–5 amino acids, *e.g*., simultaneous acetylation of K5 and K8 (H4K5acK8ac) [20–22]. H4K5acK8ac is an active chromatin mark of gene transcription [23], and disruption of the binding between H4K5acK8ac and BRDT, a testis-specific member of the BET family, disturbs spermatogenesis via transcriptional changes [24, 25]. On the other hand, BET proteins, including BRDT and BRD4, scarcely bind to H3K27ac [18–22], even though H3K27ac is extensively studied as an active chromatin mark [26, 27] to define enhancers and SEs [3, 6–11, 16, 28].

Intriguingly, a recent study identified that H3K27ac is not functionally required for transcription for genes associated with enhancers nor those with SEs in mouse embryonic stem cells [29]. Therefore, enhancer and/or SE activities may not much depend on H3K27ac but on the acetylation of other histone lysine residues. Considering the direct link between BET proteins and H4K5acK8ac [18–22], we hypothesize that high-level enrichment of H4K5acK8ac could be a better active chromatin mark than H3K27ac for defining SEs. Further, we hypothesize that genes encoding TFs that regulate a cell type–specific phenomenon, such as cancer stem-like properties, may be identified by their association with SEs with high-ranked enrichment of H4K5acK8ac, and that they may be identified by the high-level enrichment of H4K5acK8ac at the gene regulatory *cis*-elements other than SEs, *e.g*., promoters.

To test these hypotheses, we used glial cell lines related to glioblastoma multiforme (GBM) as a cell model. In this model, we examined if we could identify novel genes that regulate glioblastoma stem-like properties through the preferential enrichment of H4K5acK8ac over H3K27ac at their gene regulatory *cis*-elements. GBM is one of the most intractable brain tumors [30]; it displays intra- and inter-cellular heterogeneity, with glioblastoma cells at different stages and nontumorigenic glial progenitor cells, such as microglial cells [31, 32]. Importantly, a glioblastoma stem-like cell (GSC) in GBM contributes to cancer initiation and therapeutic resistance [33, 34]. In GSCs, treatment with JQ1, which is a small-molecule BET protein inhibitor [35, 36], or knockdown of *BRD4* induces apoptosis [37]. In addition, genes such as *ELOVL2* and *KLHDC8A*, both of which are associated with SEs defined by H3K27ac, are essential for the maintenance of GSCs [38]. Based on these findings and our above hypotheses, we considered that there may be unidentified genes involved in the regulation of glioblastoma stem-like properties that are associated with SEs defined by H4K5acK8ac in addition to those defined by H3K27ac.

Here, we compared the epigenomic profiles of H4K5acK8ac and other histone modification marks; BRD4 binding; and the transcriptomic profiles of three human glial cell lines, including a GSC line. Consistent with our hypotheses, we found that approximately half of the SEs defined by high-ranked H4K5acK8ac enrichment were distinct from those with high-ranked H3K27ac enrichment in GSCs. We demonstrated that some of the H4K5acK8ac-preferred SEs that are associated with TF-encoding genes in GSCs are involved in the maintenance of glioblastoma stem-like properties. Along with this characterization of the SEs, we identified several TF-encoding genes with H4K5acK8ac-preferred promoters involved in the maintenance of glioblastoma stem-like properties.

## Results

### Histone H4K5acK8ac is enriched at promoters and enhancers in a cell type–specific manner

**Fig. 1:**
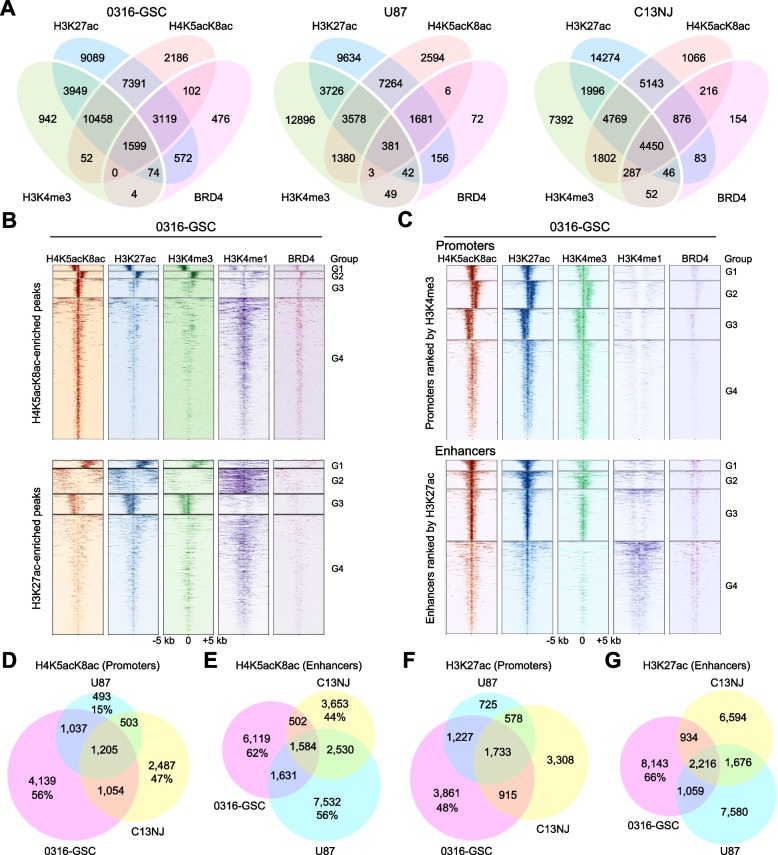
Enrichment of H4K5acK8ac at promoters and enhancers across glial cell lines. **A** Venn diagrams showing overlap of ChIP-seq peaks of H4K5acK8ac with a known enhancer mark (H3K27ac), a known promoter mark (H3K4me3), and BRD4 in 0316-GSC, U87, and C13NJ cell lines. In 0316-GSC cells, 90% of H4K5acK8ac-enriched peaks intersected with 65% of H3K27ac and 46% of H4K5acK8ac with 74% of H3K4me3. **B** and **C** Heatmaps representing different ChIP-seq datasets (H4K5acK8ac, H3K27ac, H3K4me3, H3K4me1, and BRD4) in 0316-GSC cells. Data are from within ± 5-kb from the summit of H4K5acK8ac-enriched peaks (upper, **B**) and H3K27ac-enriched peaks (lower, **B**), and promoters defined by H3K4me3 at the promoters (upper, **C**) and enhancers defined by H3K27ac located outside a promoter (lower, **C**). Each row represents a single peak. Color density indicates the average enrichment of each mark at the selected regions. H4K5acK8ac- or H3K27ac-enriched peaks and promoters or enhancers are each clustered into four groups (G1 to G4) according to the ChIP-seq profiles. **D**–**G** Venn diagrams showing overlap of ChIP-seq peaks at promoters and enhancers. H4K5acK8ac-enriched promoters (**D**) and enhancers (**E**), and H3K27ac-enriched promoters (**F**) and enhancers (**G**) intersect across the three cell lines

To understand the epigenomic landscape of glioblastoma and related glial progenitor cells, we performed chromatin immunoprecipitation followed by sequencing (ChIP-seq) of H4K5acK8ac, H3K27ac, H3K4me3, H3K4me1, and BRD4, in three human glial cell lines: a patient-derived GSC line (0316-GSC) [39], a glioblastoma line (U87), and microglia cell line (C13NJ) [40]. Using a monoclonal antibody specific to the K5- and K8-di-acetylated H4 nucleosome tail [23], we detected H4K5acK8ac peaks: (25,128 in 0316-GSC, 17,954 in U87, and 21,749 in C13NJ cells; q value < 0.01). A pairwise analysis of the five ChIP-seq datasets revealed that H4K5acK8ac peaks co-occurred with H3K27ac the most (Fig. 1A and Additional file 1: Figure S1A). This was most evident in 0316-GSC cells where 90% of H4K5acK8ac peaks co-occurred with H3K27ac peaks and 46% of H4K5acK8ac peaks co-occurred with H3K4me3 peaks (Additional file 2: Table S1). The H4K5acK8ac signal was thus highly correlated with that of H3K27ac (Pearson’s *r* = 0.85) and H3K4me3 (Pearson’s *r* = 0.80), and to a lower extent with that of H3K4me1 (Pearson’s *r* = 0.40), suggesting that the genome-wide distribution of H4K5acK8ac is most similar to that of H3K27ac among the histone modifications examined.

Next, we examined the genome-wide distribution of the five marks at H4K5acK8ac-enriched and H3K27ac-enriched regions (Fig. 1B and Additional file 1: Figure S1B). We applied *k*-means clustering in identifying four distinct groups in the ChIP-seq profiles (Groups 1 to 4 from top to bottom), within ± 5-kb from the summits of H4K5acK8ac- or H3K27ac-enriched peaks. We observed several differences in the enrichment levels of each mark on H4K5acK8ac- and H3K27ac-enriched regions. For example, in 0316-GSC cells, H3K4me3 in Group 2 had higher enrichment of the H4K5acK8ac-enriched regions than the H3K27ac-enriched regions, while H3K4me1 in Group 2 was the opposite (Fig. 1B). However, BRD4 was always strongly enriched in all the four groups of the H4K5acK8ac-enriched regions compared to the H3K27ac-enriched regions in 0316-GSC cells (Fig. 1B). These data suggest that BRD4 preferentially co-localized to the H4K5acK8ac-enriched regions in the glioblastoma stem-like cell, independent of the context of the degree of H3K4 methylation.

To understand how H4K5acK8ac is distributed at gene regulatory *cis*-elements, we defined promoters by the H3K4me3 signal (a known promoter mark) [28] and enhancers by the H3K27ac signal (a known enhancer mark) [28, 41], excluding those at promoters, and grouped the peaks using *k*-means clustering into four distinct groups (Fig. 1C and Additional file 1: Figure S1C). H4K5acK8ac and H3K27ac were enriched at both promoters and enhancers and were correlated with each other in the three cell lines. The correlation between these marks was further confirmed by immunostaining (Figure S1D, S1E; Additional file 1). We observed that immunofluorescence signals of H4K5acK8ac are well-correlated with those of H3K27ac, H3K4me3, and H3K4me1 in U87 cells.

To investigate whether the H4K5acK8ac and/or H3K27ac enrichments at promoters and enhancers were cell type–specific, we compared the corresponding ChIP-seq datasets (Fig. 1D to G). Approximately half of the H4K5acK8ac or H3K27ac enrichment either at promoters or enhancers was specific to 0316-GSC cells. Specifically, H4K5acK8ac was enriched in 4,139 promoters (56%) and 6,119 enhancers (62%) in 0316-GSC cells, compared with those in U87 cells (15% and 56%, respectively) and in C13NJ cells (47% and 44%). A similar tendency was observed for H3K27ac (Fig. 1F and G). Examples of the cell type–specific enrichment of H4K5acK8ac and H3K27ac at a promoter and enhancer in 0316-GSC cells are shown in Additional file 1: Figure S1, F to I. These data suggest that the enrichment of H4K5acK8ac is most similar to, but often remains distinctly different from, that of H3K27ac and that these histone modifications are enriched at promoters and enhancers in a cell type–specific manner.

### H4K5acK8ac is more robust upon BET inhibition and more associated with BRD4 than is H3K27ac

**Fig. 2:**
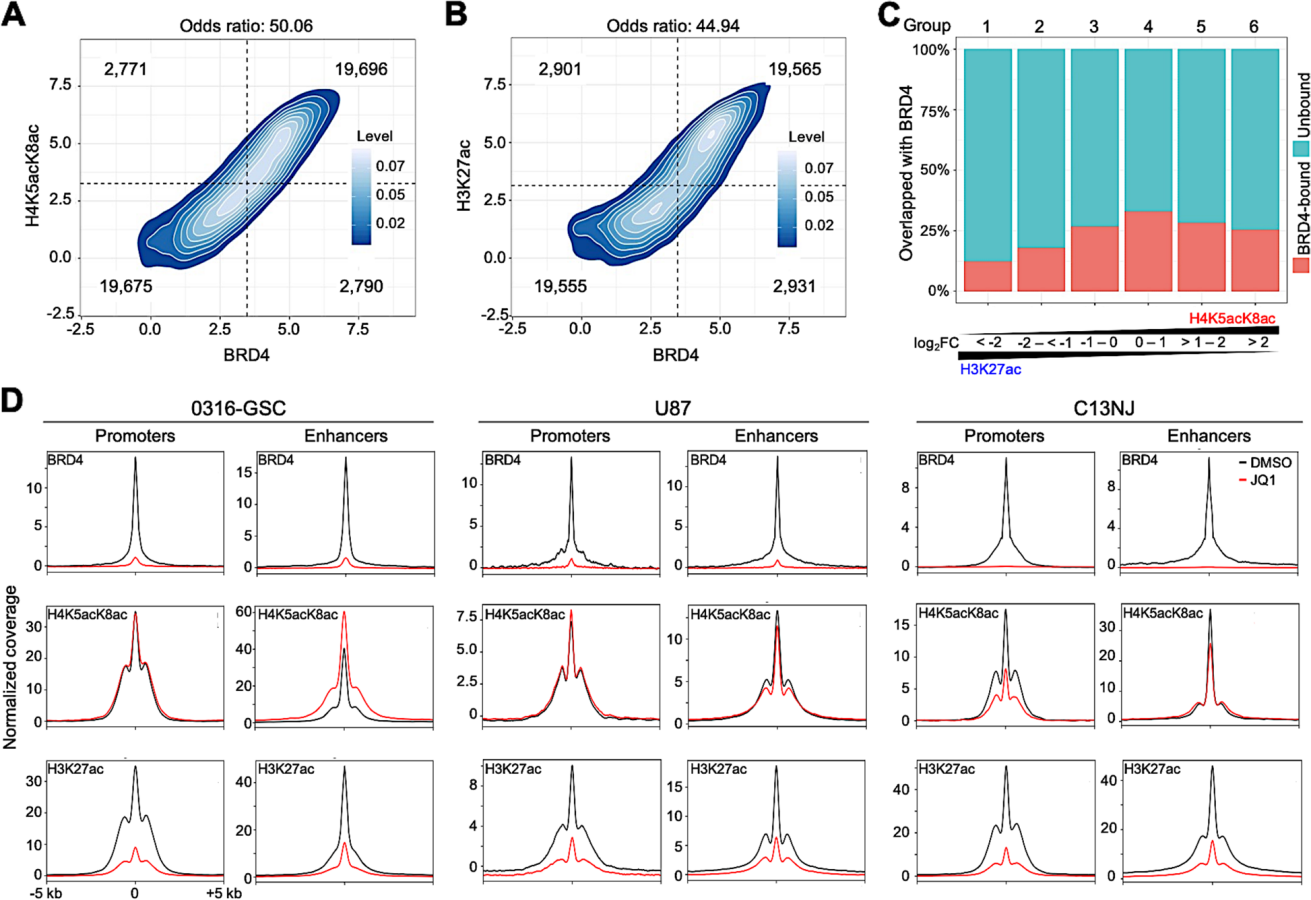
Effect of JQ1 on H4K5acK8ac-enriched regulatory regions across glial cell lines. **A** and **B** Density plots showing the association between the normalized ChIP-seq signal of BRD4 binding and H4K5acK8ac (**A**) and H3K27ac (**B**) binding. Based on the signals, the regions were categorized into four groups (separated by dotted lines); the number representing weak and strong categories of compared marks in each group is given. A higher odds ratio of binding means a higher association. A Fisher exact test was used to test whether the odds ratio was equal to 1. **C** Colocalization of BRD4 with acetylated peaks. The percentages of BRD4 ChIP-seq peaks (y-axis) that cover H4K5acK8ac- or H3K27ac-enriched peaks (x-axis) in 0316-GSC cells are shown: red bars, BRD4-bound; blue bars, BRD-unbound; FC, relative ChIP-seq signal of H4K5acK8ac over H3K27ac. **D** Effects of JQ1 on the enrichment of BRD4 (top), H4K5acK8ac (middle), and H3K27ac (bottom) at promoters and enhancers. ChIP-seq meta-profiles for dimethylsulfoxide (DMSO) (black) and JQ1-treated (red) cells represent the average read counts at reads per million (RPM) of ± 5-kb regions from the summit of BRD4-, H4K5acK8ac-, and H3K27ac-enrichment at promoters and enhancers

BRDs of BET proteins bind directly to H4K5acK8ac and scarcely to H3K27ac [18, 20, 21]. In 0316-GSC cells, BRD4 co-localization was slightly higher for H4K5acK8ac (odds ratio = 50.06; Fig. 2A) than H3K27ac (odds ratio = 44.94; Fig. 2B) at the genome-wide level. To assess whether the colocalization correlates with preferential enrichment of H4K5acK8ac over H3K27ac, we classified genomic regions with H4K5acK8ac or H3K27ac peaks into six groups based on ChIP-seq fold changes (FC) of H4K5acK8ac peak intensity over H3K27ac and vice versa*.* Promoters and enhancers were separately grouped into those that were enriched more with H3K27ac than H4K5acK8ac (Groups 1–3; log_2_FC <  − 2, − 2 to <  − 1, and − 1 to 0, respectively; hereafter, “H3K27ac-preferred; log_2_FC <  − 1”); and those enriched more with H4K5acK8ac than H3K27ac (Groups 4–6; log_2_FC 0 to 1, > 1 to 2, and > 2, respectively; hereafter, “H4K5acK8ac-preferred; log_2_FC > 1”). In 0316-GSC cells, 26% of Group 6, the most H4K5acK8ac-preferred group, were colocalized with BRD4 (Fig. 2C). On the contrary, only 13% of Group 1, the most H3K27ac-preferred group, were colocalized with BRD4, suggesting that the colocalization of H4K5acK8ac with BRD4 is twice that of H3K27ac.

Because the recruitment of RNAP II for transcription initiation is facilitated by BRD4 at TSSs [42], we examined whether H4K5acK8ac colocalizes with RNAP II more than H3K27ac by using publicly available ChIP-seq data of RNAP II from another human GSC cell line, IN528 [43]. Colocalization of H4K5acK8ac-preferred promoters with RNAP II was 2.2-fold that of H3K27ac-preferred promoters (Additional file 1: Figure S2G).

Finally, we investigated the effects of JQ1, a chemical inhibitor that detaches BET proteins from chromatin [35, 36], on the binding of BRD4 and the distribution of H4K5acK8ac and H3K27ac. Changes of the ChIP-seq levels of the above proteins at promoters and enhancers upon JQ1 treatment are shown in Fig. 2D and genome-wide changes are shown in Additional file 1: Figure S2, A to F. Across the three cell lines, upon JQ1 treatment, the binding of BRD4 at both promoters and enhancers was almost completely depleted (> 95% loss of peaks; Fig. 2D, top), and the level of H3K27ac at both promoters and enhancers was reduced in all three cell lines (Fig. 2D, bottom); the latter finding is consistent with the analysis of another glioma cell type, diffuse intrinsic pontine glioma [44]. In 0316-GSC cells, the H4K5acK8ac level was unchanged at promoters but increased at enhancers (34% of peaks); in U87 cells, it was almost unchanged at both promoters and enhancers; in C13NJ cells, it was decreased at promoters (53% of peaks) and enhancers (31% of peaks) (Fig. 2D, middle) but the decrements were less than those of H3K27ac (76% and 64% of peaks, respectively, Fig. 2D, bottom). Thus, JQ1 affected the levels of H4K5acK8ac less than those of H3K27ac, suggesting that H4K5acK8ac is more resistant to BET inhibition than H3K27ac across the three cell lines.

### Genes with the H4K5acK8ac-preferred promoters are downregulated upon BET inhibition in glioblastoma stem-like cells

**Fig. 3:**
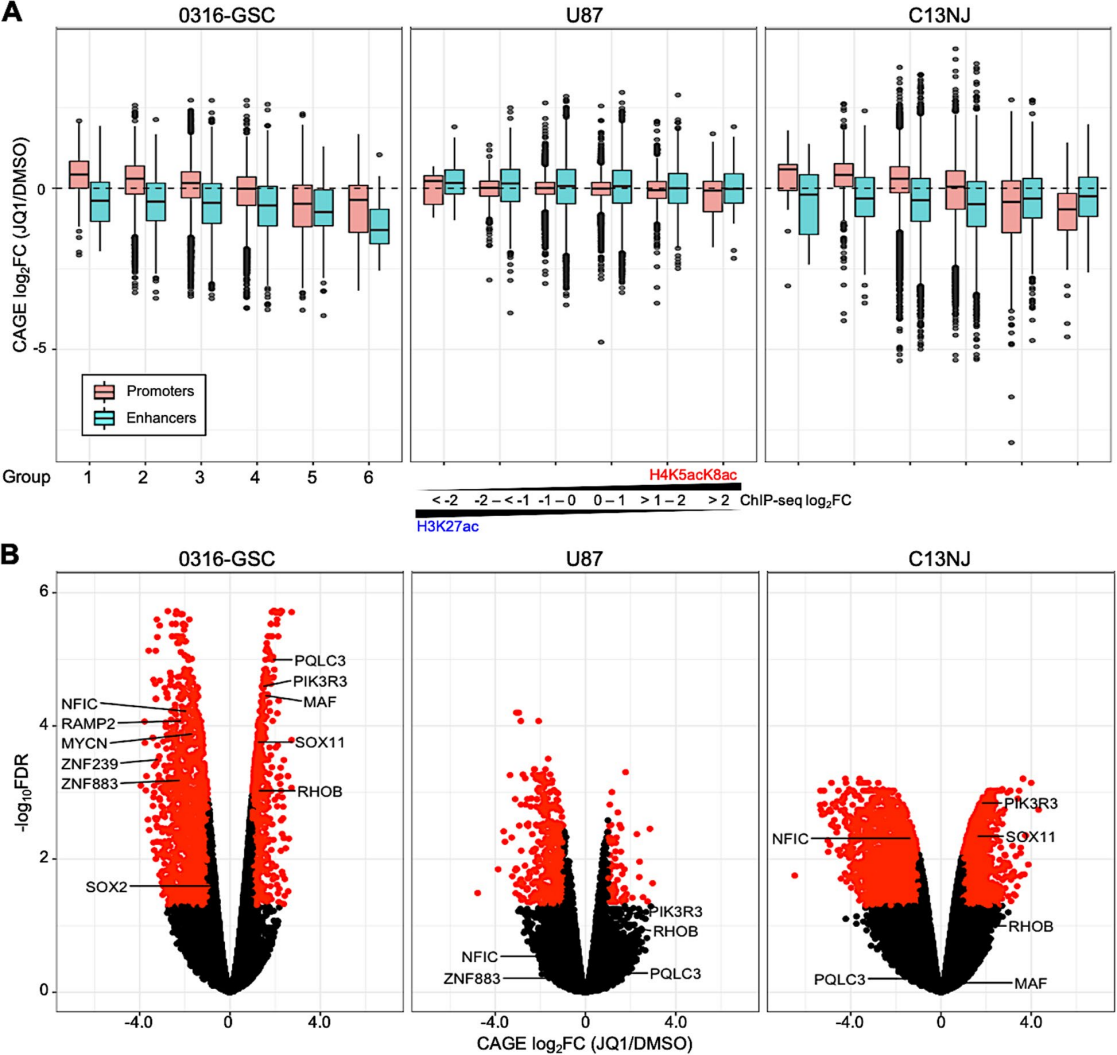
Transcriptional changes of genes with H4K5acK8ac-preferred promoters upon JQ1 treatment. **A** Relative expression of genes upon JQ1 treatment. Box plots of gene expression fold changes (FCs) for 24-h JQ1 treatment relative to DMSO (vehicle control) are shown for H3K27ac-preferred (Groups 1–3) and H4K5acK8ac-preferred (Groups 4–6; see Methods) promoters (peaks within 1-kb of the transcription start site (TSS) and enhancers (peaks > 1-kb from the TSS) in 0316-GSC, U87, and C13NJ cells (n = 2 biological replicates for each histone modification and FC value of each gene is the average of the FCs of the two biological replicates). **B** Volcano plot showing the expression differences of H4K5acK8ac-preferred differentially regulated genes in 0316-GSC cells upon JQ1 treatment. The x and y-axes show log_2_FC values and log_10_ of the false discovery rate (FDR) values, respectively. A combination of thresholds for log_2_FC values and for − log_10_FDR values are used to classify the genes as significant. Black dots represent genes that were not statistically significant

To understand the global transcriptional effects of JQ1 treatment, we profiled the transcriptomes of the three cell lines in the presence or absence of JQ1, using Cap Analysis of Gene Expression, CAGE [45] (Fig. 3). CAGE is a 5ʹ-end sequencing method that precisely quantifies transcripts from both TSSs and enhancers genome-wide [45]. In all three cell lines, with the threshold false discovery rate (FDR) set at 0.05 and log_2_FC > 0.5 or <  − 0.5, the number of downregulated genes was higher than that of upregulated genes upon JQ1 treatment (Additional file 1: Figure S3A, S3B), suggesting that JQ1 has an adverse effect on global transcription.

To understand the link between the localization of H4K5acK8ac peaks and the expression of associated genes, we next integrated the CAGE datasets with the H4K5acK8ac and H3K27ac ChIP-seq datasets for the three cell lines. Gene expression was positively correlated with levels of both H4K5acK8ac- and H3K27ac-preferred peaks genome-wide. For example, in 0316-GSC cells, gene expression was correlated with the levels of H4K5acK8ac-preferred peaks (Spearman’s correlation coefficient [*r*_*s*_] = 0.49) and H3K27ac-preferred peaks (*r*_*s*_ = 0.55). Downregulation of gene expression upon JQ1 treatment (*i.e.*, CAGE log_2_FC <  − 0.5) was most highly associated with the most H4K5acK8ac-preferred group (Group 6), especially in 0316-GSC cells (Additional file 1: Figure S3C).

Since gene transcription is regulated through a TSS-proximal promoter and a TSS-distal enhancer, we next positionally classified genomic regions into promoters and enhancers based on their location within 1-kb of the TSS of a gene or not, respectively, as in a previous study [46]. In 0316-GSC cells, gene expression was significantly positively correlated with H4K5acK8ac signals in H4K5acK8ac-preferred promoters (*r*_*s*_ = 0.55) and enhancers (*r*_*s*_ = 0.33, Fig. S3D). Genes associated with both H4K5acK8ac- and H3K27ac-preferred promoters and enhancers in 0316-GSC cells are shown in Additional file 2: Table S2. Upon JQ1 treatment, this correlation was reduced for both H4K5acK8ac-preferred promoters (*r*_*s*_ = 0.37) and enhancers (*r*_*s*_ = 0.18). Similarly, there was a positive correlation between gene expression and H3K27ac signals in H3K27ac-preferred promoters (*r*_*s*_ = 0.63) and enhancers (*r*_*s*_ = 0.36, Additional file 1: Figure S3D) in 0316-GSC cells; these correlations were slightly reduced upon JQ1 treatment (promoters, *r*_*s*_ = 0.57; enhancers, *r*_*s*_ = 0.27). Thus, a higher reduction in correlation was observed in the H4K5acK8ac-preferred promoters than in the H3K27ac-preferred promoters upon JQ1 treatment across the three cell lines. A similar tendency was observed for the enhancers to a lesser extent.

We then asked whether gene expression upon JQ1 treatment is more correlated with the preferential enrichment of H4K5acK8ac or H3K27ac at the same genomic region. Upon JQ1 treatment in 0316-GSC cells, genes with the most H4K5acK8ac-preferred promoters (Group 6) were significantly more downregulated than those with the most H3K27ac-preferred promoters (Group 1) (Welch’s *t*-test; *t* = -4.40, *P* = 5.8e^−5^; Fig. 3A); *e.g.*, the genes encoding zinc finger proteins 883 and 239 (*ZNF883* and *ZNF239*), receptor activity modifying protein 2 (*RAMP2*), and SRY-box transcription factor 2 (*SOX2*) (Fig. 3B). A similar tendency was also observed in C13NJ cells (*t* = -3.84, *P* = 3.0e^−4^), but not in U87 cells.

JQ1 treatment downregulated more genes associated with the most H4K5acK8ac-preferred enhancers (Group 6) than with the most H3K27ac-preferred enhancers (Group 1) in 0316-GSC cells (*t* = -3.87, *P* = 4.0e^−4^) but not in U87 or C13NJ cells (Fig. 3A). Examples of genes with the most H4K5acK8ac-preferred enhancers that were significantly downregulated by JQ1 treatment in 0316-GSC cells were *MYCN*, which encodes a proto-oncogenic bHLH transcription factor, and *NFIC*, which encodes nuclear factor I C (Fig. 3B). Taken together, these results suggest that downregulation of gene expression upon BET inhibition in 0316-GSC cells correlates better with H4K5acK8ac-preferred promoters than with H3K27ac-preferred promoters.

### Knockdown of genes with the H4K5acK8ac-preferred promoters reduces glioblastoma stem-like properties

**Fig. 4:**
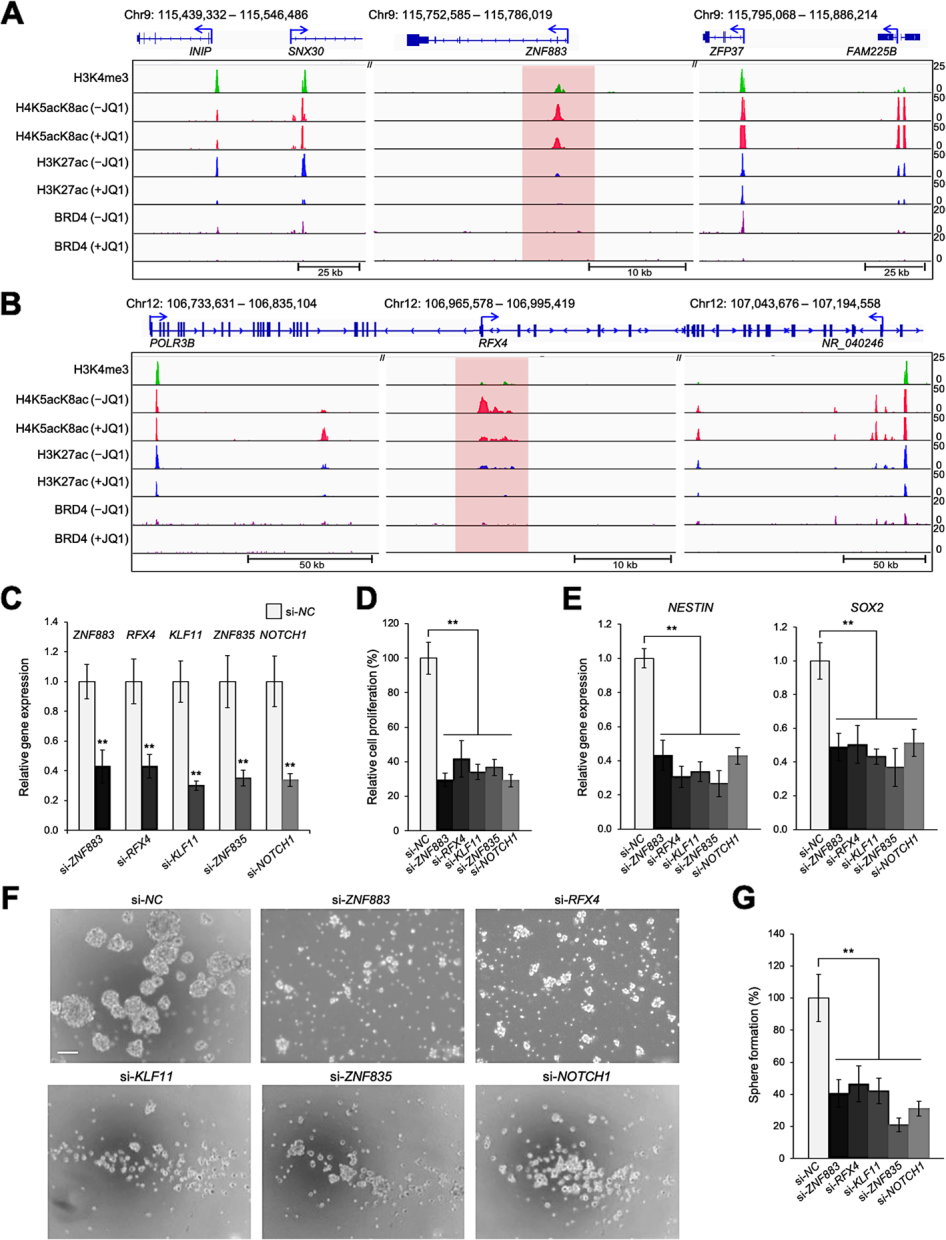
Disruption of glioblastoma stem-like properties by siRNA knockdown of genes with H4K5acK8ac-preferred promoters. **A** and **B** Comparative ChIP-seq occupancy tracks of H3K4me3, H4K5acK8ac, H3K27ac, and BRD4 at representative loci, in the presence or absence of JQ1. The promoter regions of *ZNF883* (**A**) and *RFX4* (**B**) were specifically enriched with H4K5acK8ac in 0316-GSC cells. The unique enrichment of H4K5acK8ac at promoters is highlighted in red. ChIP-seq reads were averaged from two biological replicates. **C**–**E** Disruption of gene expression by siRNA knockdown. **C** Efficiencies of siRNA knockdown of genes from Group 6 with H4K5acK8ac-preferred promoters and the GSC-specific control marker (*NOTCH1*) are compared with the negative control (*si-NC*) in 0316-GSC cells (*n* = 3). **D** Short-term proliferation assay of 0316-GSC cells subjected to siRNA knockdown. Cell proliferation rates at 7 days after siRNA knockdown of the selected genes are shown (*n* = 6). **E** Expression of stem cell marker genes, *NESTIN* and *SOX2*, following siRNA knockdown of the selected genes (*n* = 3). **F** and **G** Sphere formation assay following siRNA knockdown of the selected genes. **F** Phase-contrast images of 0316-GSC cells treated with target-specific siRNA. Images are representative of three independent experiments. Scale bar, 50 μm. **G** In vitro sphere formation efficiency of 0316-GSC cells treated with siRNA for 2 weeks (*n* = 3). **C**–**E** and **G** Data are means ± SEM **P < 0.01 (two-tailed Student’s *t*-test)

Because TFs regulate stem-like properties in some cancers [47], we set out to identify TFs that might function as master regulators of glioblastoma stem-like properties. Using the PANTHER (Protein Analysis Through Evolutionary Relationships) Database [48], we identified candidate genes encoding TFs and transcription coregulators from among the genes associated with the first- and second-most H4K5acK8ac-preferred promoters (Groups 5 and 6) in 0316-GSC cells (Additional file 1: Figure S4A, S4E): *e.g.*, *ZNF883* (Fig. 4A)*,* regulatory factor X4 (*RFX4,* Fig. 4B)*,* Krüppel-like factor 11 (*KLF11*)*,* and *ZNF835*.

We next analyzed the enrichment of gene ontology (GO) terms in the list of JQ1-downregulated genes (*i.e.*, CAGE log_2_FC <  − 0.5) with H4K5acK8ac-preferred promoters by using Gene Set Enrichment Analysis, GSEA [49] and Enrichr [50], respectively (Additional file 1: Figure S4F and Additional file 2: Table S3). The identified TF candidate genes with H4K5acK8ac-preferred promoters, *i.e.*, *ZNF883*, *RFX4*, *KLF11*, and *ZNF835*, were all significantly over-represented in the GO molecular function of the RNAP II regulatory region-specific DNA binding activity (GO:0000977; Additional file 1: Figure S4G and Additional file 2: Table S4), suggesting the involvement of these genes in transcriptional regulation of 0316-GSC cells.

To investigate whether these genes with H4K5acK8ac-preferred promoters regulate glioblastoma stem-like properties, *i*.*e*., marker gene expression for stem cells and sphere formation efficiency [51, 52], we disrupted *ZNF883*, *RFX4, KLF11*, and *ZNF835* by siRNA knockdown in 0316-GSC cells. The siRNA knockdown of these genes or the positive control gene *NOTCH1* [53] in 0316-GSC cells (Fig. 4C) reduced the cell proliferation rate by 70%, 59%, 66%, 63%, or 70% respectively, compared with the negative control (si-NC) (all *P* < 0.01, Fig. 4D). siRNA knockdown of these genes significantly reduced the expression of the marker genes for stem cells, *NESTIN* and *SOX2* in 0316-GSC cells (both *P* < 0.01, Fig. 4E), but not the expression of *NESTIN* in U87 cells or that of *c-MYC*, a stemness-associated gene, in C13NJ cells (Additional file 1: Figure S4B, S4C). The specific knockdown of a JQ1-insensitive gene, *ZNF518B*, did not reduce the expression of *NESTIN* or *SOX2* in 0316-GSC cells (Additional file 1: Figure S4D). Sphere formation efficiency, a measure of stem-like properties, is an independent predictor of glioma tumor progression [51]. We observed that siRNA knockdown of *ZNF883*, *RFX4*, *KLF11*, or *ZNF835*, or the positive control *NOTCH1* significantly reduced sphere formation efficiency in 0316-GSC cells (all *P* < 0.01; Fig. 4F and G). We propose that these four genes with H4K5acK8ac-preferred promoters contribute to the proliferation and stem-like properties of 0316-GSC cells.

### Identification of SEs by top-ranked H4K5acK8ac signal

**Fig. 5:**
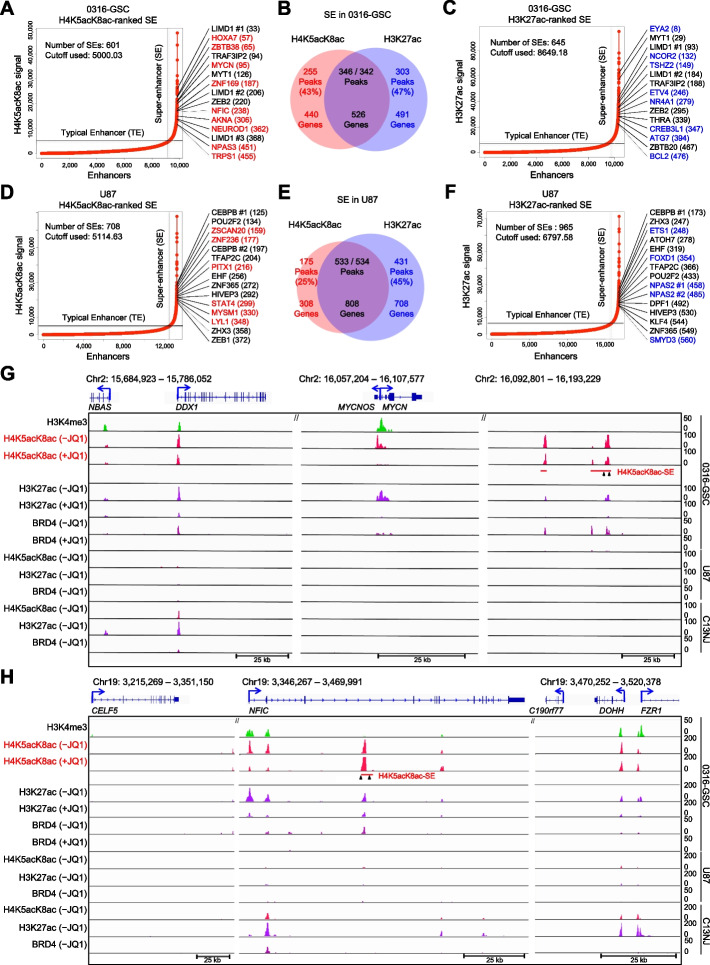
Defining super-enhancers by H4K5acK8ac enrichment ranking. Enhancers in 0316-GSC (**A**–**C**) and U87 (**D**–**F**) cells were ranked by H4K5acK8ac or H3K27ac signal level by using the ROSE algorithm; those with extremely high signals were defined as super-enhancers (SEs) (see Methods for threshold calculations). JQ1-downregulated transcription factor candidate genes with H4K5acK8ac- (red) or H3K27ac-preferred (blue) SEs are shown. Venn diagrams show the number of peaks and associated genes with H4K5acK8ac- or H3K27ac-preferred SEs or both (the intersection, purple) for each cell line. **G** and **H** Representative ChIP-seq occupancy tracks of genes with H4K5acK8ac-preferred SEs (*MYCN* and *NFIC*) in 0316-GSC, U87, and C13NJ cells. Arrowheads under the SE bar show the position for CRISPR-Cas9–mediated deletion of the SE region. ChIP-seq reads were averaged from two biological replicates

SEs comprise multiple typical enhancers (TEs) in close genomic proximity [3, 6, 7]; SEs are highly enriched in the active chromatin mark H3K27ac [3] and transcriptional coactivators, *e.g.*, Mediator and BRD4 [6, 7]. Since high-level H3K27ac enrichment is predictive of an SE [3], we hypothesized that high-level enrichment of H4K5acK8ac could also be predictive of an SE and might identify SEs missed by H3K27ac signal ranking. To define SEs based on H4K5acK8ac signal enrichment across the three cell lines, we used the Rank Ordering of Super Enhancer (ROSE) [6, 7] algorithm (Fig. 5A and D). Using GREAT [54], we analyzed the SEs obtained in the three cell lines with which genes they are associated (*i.e.*, associated genes). We then compared the genes associated with SEs identified by H4K5acK8ac to those identified by H3K27ac (Fig. 5A to F and Additional file 1: Figure S5A). In 0316-GSC cells, 43% of H4K5acK8ac-ranked SEs were not detected as H3K27ac-ranked SEs and were designated as H4K5acK8ac-preferred SEs; 57% were also listed as H3K27ac-ranked SEs (Fig. 5B). Similarly, 25% of the H4K5acK8ac-ranked SEs in U87 cells (Fig. 5E) and 7% of those in C13NJ cells (Additional file 1: Figure S5A, middle) were unique to H4K5acK8ac. In 0316-GSC cells, 255 H4K5acK8ac-preferred SEs were associated with 440 genes and 303 H3K27ac-preferred SEs were associated with 491 genes (Fig. 5B and Additional file 2: Table S5). In contrast, the proportions of H4K5acK8ac-preferred TEs were similar in 0316-GSC (26%), U87 (27%), and C13NJ (21%) (Additional file 1: Figure S5B).

Since gene transcription is regulated by enhancers, we investigated whether gene transcription upon JQ1 treatment is correlated with the preferential enrichment of H4K5acK8ac or H3K27ac at enhancers. We observed that JQ1 treatment resulted in a greater downregulation of genes associated with the H4K5acK8ac-preferred enhancers compared to the H3K27ac-preferred enhancers in 0316-GSC cells (*P* = 6.0e^−12^, Additional file 1: Figure S5C), but not in U87 or C13NJ cells (Fig. 3A). The genes associated with the H4K5acK8ac-preferred SEs were highly cell type–specific: 95% (418/440) of those associated with H4K5acK8ac-preferred SEs and 69% (339/491) of those associated with H3K27ac-preferred SEs were specifically expressed in 0316-GSC cells (Additional file 1: Figure S5D, S5E) and both groups of genes were identified with their classification by using the Drug-Gene Interaction Database [55] (Additional file 1: Figure S5F, S5G). The cell type–specific SEs at *MYCN* (Fig. 5G) and *NFIC* (Fig. 5H) are specifically enriched with H4K5acK8ac in 0316-GSC cells but not in U87 or C13NJ cells. Collectively, these observations imply that H4K5acK8ac could be used as an indicator of SEs that are distinct from those detected by H3K27ac.

Since SEs are highly associated with TF-encoding genes [6, 7] and some of the TF-encoding genes associated with the H4K5acK8ac-preferred SEs may regulate glioblastoma stem-like properties, we identified TF candidate genes using the PANTHER database [48] and examined their downregulation upon JQ1 treatment. The top 15 TF candidate genes associated with H4K5acK8ac- and H3K27ac-ranked SEs (*i.e.*, the high rank of ChIP-seq signal) in each cell type whose expression is downregulated upon JQ1 treatment (*i.e.*, CAGE log_2_FC <  − 0.5) are shown in Fig. 5 and Additional file 1: Figure S5A. GO molecular functions and GO biological pathways of the genes associated with the H4K5acK8ac- and H3K27ac-preferred SEs and TEs are separately shown in Additional file 1: Figure S5, H to K. Top-ranked TF candidate genes with H4K5acK8ac-preferred SEs in 0316-GSC cells, *e.g.*, homeobox A7 (*HOXA7*), *MYCN*, and *NFIC*, are included in the several GO terms annotated by Enrichr (Additional file 2: Table S4). For example, they are included in the RNAP II regulatory region-specific DNA binding activity (GO:0000977; Additional file 1: Figure S5H). Also, *MYCN* and *NFIC* are included in the cell cycle pathway and the *PLK1* activity at the G2/M transition pathway, respectively (Additional file 1: Figure S5I), suggesting their potential contribution to the maintenance of proliferation and/or stem-like properties of GSCs.

### Deletion of H4K5acK8ac-preferred SEs reduces the expression of associated genes in GSCs

**Fig. 6:**
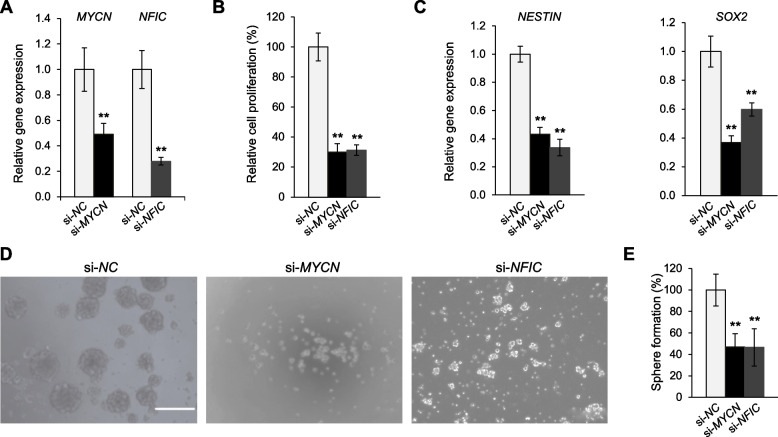
Disruption of glioblastoma stem-like properties by siRNA knockdown of genes with H4K5acK8ac-preferred super-enhancers. **A**–**C** Disruption of genes by siRNA knockdown. **A** siRNA knockdown of *MYCN* and *NFIC* compared with the negative control (*si-NC*) in 0316-GSC cells (*n* = 3). **B** Short-term proliferation assay of 0316-GSC cells subjected to siRNA knockdown. Cell proliferation rates at 7 days after siRNA knockdown of *MYCN* and *NFIC* are shown (*n* = 6). **C** Expression of stem cell marker genes, *NESTIN* (left) and *SOX2* (right), following siRNA knockdown of *MYCN* and *NFIC* (*n* = 3). **D** Sphere formation assay. Phase-contrast images of 0316-GSC cells treated with *si-MYCN*, *si-NFIC*, or *si-NC* for 2 weeks. Images are representative of three independent experiments. Scale bar, 50 μm. **E** Quantitation of sphere formation results for 0316-GSC cells (*n* = 3). **A**–**C** and **E** Data are means ± SEM **P < 0.01 (two-tailed Student’s *t*-test)

To investigate whether *MYCN* or *NFIC* are involved in the maintenance of glioblastoma stem-like properties as predicted, we assessed the effects of their siRNA knockdown on 0316-GSC cells. Knockdown of either gene (Fig. 6A) reduced the cell proliferation rate by 70% and 69%, respectively (both *P* < 0.01, Fig. 6B); reduced the expression of stem cell marker genes, *NESTIN* (*si-MYCN*, 57%; *si-NFIC*, 66%) and *SOX2* (*si-MYCN*, 63%; *si-NFIC*, 42%; Fig. 6C); and significantly decreased the sphere formation rate (*P* < 0.01, Fig. 6D and E).

**Fig. 7:**
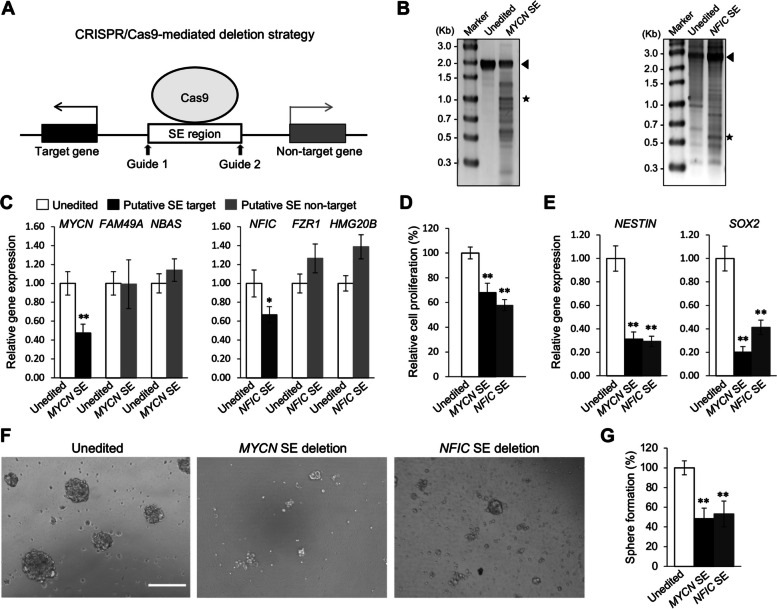
Genes associated with H4K5acK8ac-preferred super-enhancers are involved in the glioblastoma stem-like properties. **A** Schematic representation showing the CRISPR-Cas9–mediated genome editing approach for SEs. Guide indicates gRNA. **B** Deletion of the H4K5acK8ac-preferred SEs associated with *MYCN* and *NFIC* in 0316-GSC cells. Expected band sizes of genomic DNA for unedited (arrowhead) and SE-edited samples (asterisk) are marked. Images of the uncropped gel are shown in Figure S9A. **C**–**G** Biological effects of deletion of the H4K5acK8ac-preferred SEs associated with *MYCN* and *NFIC* in 0316-GSC cells. **C** Quantitative reverse-transcription PCR analysis of the expression of *MYCN* and *NFIC* and non-target genes following SE deletion (*n* = 3). **D** Cell proliferation rates at 4 days after SE deletion (*n* = 4). **E** Expression of stem cell marker genes, *NESTIN* (left) and *SOX2* (right), at 4 days after SE deletion (*n* = 3). **F** Phase-contrast images of 0316-GSC cells at 14 days after SE deletion. Images are representative of three independent experiments. Scale bar, 50 μm. **G** In vitro sphere formation efficiency of 0316-GSC cells at 2 weeks after SE deletion (*n* = 3). **C**–**E** and **G** Data are means ± SEM *P < 0.05, **P < 0.01 (two-tailed Student’s *t*-test)

To understand whether the H4K5acK8ac-preferred SEs regulate expression of the associated genes in 0316-GSC cells, we performed CRISPR-Cas9–mediated SE deletion (Fig. 7 and Additional file 1: Figure S6 to S8). Regions with ChIP-seq log_2_FC (H4K5acK8ac/H3K27ac) scores of 1.58 and 1.20 in the SE regions of *MYCN* and *NFIC*, respectively, were chosen as validation targets for CRISPR-Cas9. Since there were no H4K5acK8ac-preferred SEs devoid of H3K27ac peak signals in top 20 TF-encoding genes (Fig. 5A), we also selected another peak in the H4K5acK8ac-preferred SE of homeobox protein Hox-A7 (*HOXA7*) with one of the least H3K27ac peak signals among the 20 genes (ChIP-seq log_2_FC (H4K5acK8ac/H3K27ac) score of 2.06). Paired guide RNAs (gRNAs) were chosen that flank the target regions in the H4K5acK8ac-preferred SEs (Additional file 1: Figure S6A, S7B and Additional file 2: Table S6). We confirmed that the targeted SE regions were excised from the genome in the gRNA-treated cells (Fig. 7B and Additional file 1: Figure S7D); as a control, cells were treated with a computationally validated negative control gRNA (designated unedited).

The CRISPR-Cas9–mediated deletion of a targeted H4K5acK8ac-preferred SE (*i.e.*, 980-bp for *MYCN*, 2,232-bp for *NFIC,* or 1,962-bp for *HOXA7*) resulted in significant downregulation of *MYCN* (53%), *NFIC* (34%), and *HOXA7* (35%), respectively, compared with the unedited cells (Fig. 7C and Additional file 1: Figure S7E). Although the editing efficiency for *NFIC* SE was the lowest (19%, Fig. 7B, right), we found a reduction in relative gene expression of *NFIC* (34%, Fig. 7C). These deletions did not change the expression of the putative non-target genes, *FAM49A* and NBAS subunit of NRZ tethering complex (*NBAS*), which are located 702-kb downstream and 475-kb upstream of *MYCN*, respectively, *FZR1* and *HMG20B*, which are located 105-kb and 180-kb downstream of *NFIC*, respectively, or src kinase associated phosphoprotein 2 (*SKAP2*), which is located 293-kb downstream of *HOXA7* (*P* > 0.05; Fig. 7C and Additional file 1: Figure S7E). While the deletion of the H4K5acK8ac-preferred SE of *HOXA7* reduced the expression of a putative non-target gene, *HOXA9,* which is located 8.95-kb upstream of *HOXA7*, this reduction was not significant (Additional file 1: Figure S7E). These results suggest that the examined H4K5acK8ac-preferred SE regions are involved in the regulation of the target gene expression in 0316-GSC cells. However, our study does not preclude the possibility of the colocalized minor H3K27ac peaks being involved in the function of an enhancer or SE.

### Deletion of H3K27ac-preferred SEs has less effect to that of H4K5acK8ac-preferred SEs

Next, we analyzed the effect of deleting H3K27ac-preferred SEs in 0316-GSC cells; *BCL2* and *ZBTB7B* were chosen as targets (Additional file 1: Figure S8, A to D). These SEs were selected by the criterion of low signal of the H4K5acK8ac peaks among the top 20 TF-encoding genes (Fig. 5C). The ChIP-seq log_2_FC (H4K5acK8ac/H3K27ac) scores for the selected peaks in the SE regions at *BCL2* and *ZBTB7B* are -0.64 and -0.62, respectively. Genome editing efficiency for the *BCL2* and *ZBTB7B* SE region in 0316-GSC cells was 34% and 57%, respectively (Figure S8F; Additional file 1). The CRISPR-Cas9–mediated deletion of H3K27ac-preferred SEs (*i.e.*, 694-bp for *BCL2* or 898-bp for *ZBTB7B*; Figure S8C, S8D; Additional file 1) did not reduce the expression of both the putative target and putative non-target genes (Figure S8G; Additional file 1). While we only investigated a limited number of H3K27ac-preferred SEs, our findings suggest that the H3K27ac peaks in these SE regions do not play a role in the transcriptional regulation of the associated genes.

### Deletion of H4K5acK8ac-preferred SEs decreases stem-like properties in GSCs

Finally, we investigated whether the H4K5aK8ac-preferred SE regions (*i.e.*, at *MYCN* or *NFIC*) or the H3K27ac-preferred SE regions (*i.e.*, at *BCL2* or *ZBTB7B*) are involved in maintaining stem-like properties in 0316-GSC cells, such as stem cell marker gene expression and sphere formation. Deletion of either of the *MYCN* or *NFIC* SE region significantly decreased the proliferation of 0316-GSC cells compared with unedited cells (both *P* < 0.01, Fig. 7D). It significantly reduced the expression of the stem cell marker genes *NESTIN* and *SOX2* (both *P* < 0.01, Fig. 7E), and decreased the sphere formation efficiency (both *P* < 0.01, Fig. 7F and G) in these cells. To the contrary, deletion of the H3K27ac-preferred SE regions of *BCL2* and *ZBTB7B* did not significantly decrease the rate of cell proliferation, the expression of stem cell marker genes, or the sphere formation efficiency in 0316-GSC cells (Figure S8H to K; Additional file 1). Together these data suggest that the H4K5acK8ac-preferred SE regions associated with either *MYCN* or *NFIC* are involved in the regulation of the stem-like properties of 0316-GSC cells whereas the examined H3K27ac-preferred SE regions are not.

## Discussion

Most earlier studies of histone acetylation marks focus on H3K27ac as a mark of active promoters, enhancers, and SEs [3, 28, 41]. However, a recent study demonstrated that the depletion of H3K27ac at enhancers and SEs does not affect enhancer activity and gene transcription [29]. Therefore, using human glial cell lines as models, we attempted to define SEs by focusing on H4K5acK8ac, to which the BET proteins bind stronger than H3K27ac, and thereby identify genes involved in cancer stem-like properties. We identified groups of active promoters, enhancers, and SEs that were preferentially enriched with H4K5acK8ac over H3K27ac at a single-nucleosome resolution (Figs. 4–7). Supporting our hypotheses, we revealed the existence of cell type–specific H4K5acK8ac-preferred SEs and their associated genes, at least some of which were associated with the maintenance of glioblastoma stem-like properties (Figs. 5–7). Importantly, the present approach focusing on the high-level enrichment of H4K5acK8ac enabled us to identify a group of functionally active SEs that were missed by profiling of H3K27ac alone (Fig. 5B, E and Additional file 1: Figure S5A, middle).

Given that 90% of H4K5acK8ac peaks had H3K27ac signals in GSCs, there is a possibility that these two histone acetylation marks are virtually the same. However, we found that 43% of the H4K5acK8ac-ranked SEs did not overlap with SEs ranked by H3K27ac in GSCs (Fig. 5B), suggesting that almost half of the H4K5acK8ac-ranked SEs are formed independently from H3K27ac-ranked SEs. By conducting CRISPR-Cas9–mediated genome editing of SE regions, we revealed that certain H4K5acK8ac-preferred SEs were responsible for the expression of associated genes (*i.e*., *MYCN* and *NFIC*) in GSCs (Fig. 7A to C) and the maintenance of glioblastoma stem-like properties (Fig. 7F and G). Interestingly, the deletion of the H3K27ac-preferred SE regions of *BCL2* and *ZBTB7B* neither reduced the expression of their target genes nor the glioblastoma stem-like properties (Figure S8; Additional file 1). It should be noted, however, that the efficiency of the genome edition in this study was 19% at its lowest (for *NFIC* SE), which may not be sufficient to validate the effectiveness of SE removal. In addition, it is important to validate the direct link between the SEs and their target genes using a 3D genomic interaction analysis method, such as chromosome conformation capture coupled with next-generation sequencing (Hi-C) [56] and Hi-C followed by chromatin immunoprecipitation (HiChIP) [57]. Further studies are needed to gain a comprehensive understanding of the roles of the H3K27ac- and H4K5acK8ac-preferred SEs in the regulation of glioblastoma stem-like cells or other cellular contexts.

We identified a group of TF candidate genes with H4K5acK8ac-preferred promoters (*e.g*., *ZNF883* and *RFX4*) and showed that they were also involved in the maintenance of glioblastoma stem-like properties (Fig. 4). Interestingly, JQ1 downregulated more TF candidate genes with H4K5acK8ac-preferred promoters (*e.g.*, *ZNF883*, *RFX4*, *KLF11*, *ZNF835*, *STAT5A*, *HNF4G*, and *NFATC4*) than with H3K27ac-preferred promoters. Since BET proteins preferentially associate with H4K5acK8ac rather than H3K27ac, H4K5acK8ac at promoters may be more involved in BET protein–dependent transcriptional regulation than H3K27ac. Thus, collectively, this study demonstrates that H4K5acK8ac at promoters and SEs makes a hitherto unexplored contribution to transcriptional regulation linked to the glioblastoma stem-like properties.

H4K5acK8ac signals were robust to JQ1 treatment and behaved differently from JQ1-sensitive H3K27ac and BRD4 signals (Fig. 2D). It is intriguing as to why the level of H4K5acK8ac upon BET inhibition was more robust than that of H3K27ac. Since BET proteins (*e.g*., BRD4) directly bind to H4K5acK8ac and scarcely to H3K27ac [20–22, 58], H4K5acK8ac would be expected to become more exposed to histone deacetylases upon removal of BET proteins by JQ1. However, as was observed in H23 cells [23], H4K5acK8ac levels were not reduced as much as H3K27ac levels upon JQ1 treatment across the three glial cell lines; even a slight increase was observed for the enhancers in 0316-GSC (Fig. 2). A possible explanation could involve 1) non-BET bromodomain–containing proteins, 2) a protein complex associated with the BET family proteins, and/or 3) the dynamics of histone (de)acetylation.

For the first possibility, a non-BET BRD-containing protein, TATA box–binding protein-associated factor 1 (TAF1), preferentially binds to multi-acetylated lysines of the histone H4 tail through its double BRDs [59]. Therefore, erasure of H4K5acK8ac might be prevented by such non-BET BRD-containing protein binding to H4K5acK8ac in the presence of JQ1. For the second possibility, JQ1 neither displaces BRD4 from chromatin nor alters H3K27ac level in BET inhibitor–resistant breast cancer cells [60]. This is presumably because when BRD4 is hyperphosphorylated, it recruits MED1 which in turn helps BRD4 associate with the acetylated chromatin [60]. Thus, such a protein complex associated with the BET family proteins at the acetylated chromatin may prevent the erasure of H4K5acK8ac upon BET inhibition. Thirdly, histone acetyltransferases such as p300/CBP [61] and Tip60 [62] may contribute to the maintenance of the H4K5acK8ac level. Alternatively, histone deacetylases may not deacetylate multi-acetylated histone H4 tails (*e.g.*, H4K5acK8ac) well compared with mono-acetylated histone tails (*e.g.*, H4K5ac, H4K8ac, H3K27ac) because of charge differences and substrate preferences [63]. A comprehensive pharmacologic intervention utilizing inhibitors against BET proteins, non-BET proteins, histone acetyltransferases, and histone deacetylases, may help reveal the mechanism governing the robustness of H4K5acK8ac linked to transcriptional regulation.

H4K4acK8ac may contribute to transcriptional regulation through the cooperation of a TF, a chromosome architectural protein, or both. Regarding TFs, binding sequences of SOX9, OCT4, NFIC, SCL, and NFATC2 were detected in a significant manner in the genomic regions within 500-bp of the H4K5acK8ac-ranked SEs in GSCs (Additional file 1: Figure S5L, left). Some TFs, *e.g.*, RUNX1 and MYB, establish auto-regulatory networks for transcriptional regulation through binding to SEs associated with *RUNX1* and *MYB,* respectively [64, 65]. Interestingly, there are two predicted MYCN-binding sequences (*e.g.*, CATTTG) within the 980-bp CRISPR-Cas9–mediated SE-deleted region (Additional file 1: Figure S6A) of the H4K5acK8ac-preferred *MYCN* SE. Therefore, a TF such as MYCN may activate its gene expression in a positive feedback manner by binding to its H4K5acK8ac-preferred SE.

Regarding cooperation of a chromosome architectural protein, H4K5acK8ac may facilitate chromatin looping between enhancers/SEs and promoters, which is possibly mediated by a protein such as CCCTC-binding factor (CTCF) and Yin Yang 1 (YY1). CTCF contributes to the formation of a chromatin loop structure by interacting with two separate chromatin domains, thereby controlling gene expression [66]. Chromatin looping between enhancers and promoters is mediated by CTCF and cohesin [67], and by YY1 [68]. Intriguingly, in GSCs, there was a genome-wide correlation between the enrichment of H4K5acK8ac and those of CTCF [69] and YY1 [70] (Pearson correlation, *r* = 0.25 and 0.27, respectively). Therefore, integrating Hi-C and/or HiChIP of H4K5acK8ac with those of CTCF and YY1 may reveal a role for H4K5acK8ac in the formation of the chromatin looping between enhancers/SEs and promoters.

## Conclusions

In summary, we have demonstrated that in addition to H3K27ac, H4K5acK8ac is a key histone acetylation mark effective in a promoter- and enhancer/SE-mediated transcriptional regulation. CRISPR-Cas9–mediated genetic ablation revealed that the SEs that are preferentially enriched with H4K5acK8ac over H3K27ac in the glioblastoma stem-like cell line are involved in the transcriptional regulation of associated genes, thereby maintaining the glioblastoma stem-like properties. Our results highlighted that H4K5acK8ac is an indicator of functional SEs and can be used to detect SEs that are missed by the H3K27ac-enrichment ranking. The present approach of defining SEs by histone H4 multi-acetylation may be used to identify novel key TFs regulating cell-type specificity in a variety of cellular models.

## Methods

### Cell culture and drug treatment

Human glioblastoma stem-like cell line (0316-GSC) [39] was maintained in neurobasal medium (Life Technologies) containing N2, B27 (Life Technologies), and human recombinant FGF2 (10 ng/ml; 233-FB-025) and EGF (20 ng/ml; 236-EG-200) (R&D Systems). The U87 cell line (ATCC HTB-14) was obtained from the American Type Culture Collection, and maintained in Eagle’s minimum essential medium (MEM) supplemented with 10% fetal bovine serum (FBS), 100 units/ml penicillin, and 10 μg/ml streptomycin. The human microglia cell line C13NJ was established previously [40, 71], and was maintained in DMEM supplemented with 10% FBS and 50 μg/ml gentamicin. Twenty-four hours before drug treatment, 0316-GSC, U87, and C13NJ cells were cultured on a 100 mm plate at 2.5 × 10^6^, 2 × 10^6^, and 2 × 10^6^ cells/plate. Then all cell lines were treated with 5 μM JQ1 (2070–1, BioVision) dissolved in DMSO or DMSO (vehicle control) for 24 h. Medium containing JQ1 or an equivalent amount of DMSO was prepared for each cell line on the day of treatment.

### Antibodies

Antibodies used were as follows: anti-H4K5acK8ac (mouse monoclonal) [23], anti-H3K4me3 (rabbit monoclonal, 17–614, Millipore), anti-H3K4me1 (rabbit polyclonal, C15410194, Diagenode), anti-H3K27ac (rabbit polyclonal, ab4729, Abcam), and anti-BRD4 (rabbit polyclonal, A301-985A100, Bethyl Laboratories).

### Chromatin immunoprecipitation

0316-GSC cells were treated with 0.5% (v/v) formaldehyde for 6 min at room temperature to crosslink histone to DNA; this was followed by the addition of 1.25 M glycine to quench the crosslinking reaction. Cells were subsequently washed with ice-cold phosphate-buffered saline (PBS) containing bovine serum albumin (BSA; 5 mg/ml), ice-cold PBS alone, and PBS with protease inhibitors. After aspiration of the supernatant, cell pellets were flash-frozen in liquid nitrogen and stored at − 80 ℃. Cell pellets were lysed with lysis buffer (50 mM Tris–HCl pH 8.1 containing 1% sodium dodecyl sulfate (SDS), 10 mM ethylenediaminetetraacetic acid (EDTA), and cOmplete Protease Inhibitor Cocktail (4,693,116,001, Roche) by incubation on ice for 10 min, followed by sonication (Covaris S220 sonicator: duty cycle, 5%; cycles per burst, 200; volume, 130 μl) for 8 min on low cell chromatin shearing mode. The sheared chromatin was diluted in 20 mM Tris–HCl pH 8.1 containing 150 mM NaCl, 1% Triton X-100, and 2 mM EDTA. An aliquot of sheared chromatin was treated with RNase A and proteinase K (both 20 mg/ml), de-crosslinked by heating (65 ℃ for 5 to 6 h), and used as input control. The sheared chromatin was incubated for 10–12 h at 4 °C with antibody-bound magnetic beads (40 μl of Dynabeads sheep anti-mouse IgG, Cat. no. 11201D or sheep anti-rabbit IgG, Cat. no. 11203D) for each ChIP. Anti-H4K5acK8ac (3 μg for 4 × 10^6^ cells), anti-H3K4me3 (3 μg for 3 × 10^6^ cells), anti-H3K27ac (2.5 μg for 3 × 10^6^ cells), anti-H3K4me1 (4 μg for 4 × 10^6^ cells), and anti-BRD4 (10 μg for 8 × 10^6^ cells) antibodies were used for each ChIP. The antibody-bead conjugated chromatin was washed twice with low-salt wash buffer (20 mM Tris–HCl pH 8.1 containing 150 mM NaCl, 0.1% SDS, 2 mM EDTA, and 1% Triton X-100), twice with high-salt wash buffer (the above buffer, except containing 500 mM NaCl), twice with LiCl wash buffer (10 mM Tris–HCl pH 8.1 containing 0.25 M LiCl, 1% deoxycholic acid, and 1 mM EDTA) and twice with Tris–EDTA wash buffer (10 mM Tris–HCl pH 8.1 containing 1 mM EDTA). For BRD4-ChIP, single sequential washes of low-salt, high-salt, LiCl wash buffer and a double wash of Tris–EDTA wash buffer were used. The chromatin was eluted in 100 mM NaHCO_3_ containing 1% SDS and de-crosslinked at 65 ℃ for 5 to 6 h. RNA and protein were digested using 1 μl of RNase A and 1.5 μl of Proteinase K (both 20 mg/ml). The DNA was then isolated using Agencourt AMPure XP beads (A63880, Beckman Coulter) for 0316-GSC cells, or a MinElute column (Qiagen) for U87 and C13NJ cells. DNA was then subjected to quantitative PCR (qPCR) with locus-specific primers. The ChIP-qPCR results were normalized to the amount of input DNA. Two biological replicates were used for each histone modification and BRD4. For ChIP-seq library preparation, a NuGEN R110-LC Mondrian workstation (2 ng of ChIP DNA and 11 PCR cycles) was used, and each ChIP-seq library was subjected to 50-bp sequencing using a HiSeq 2500 system (Illumina).

### ChIP-seq data analysis

Mapping of ChIP-seq data was performed by aligning reads to the hg19 human genome assembly using bowtie2 [72]. All datasets were processed by AQUAS transcription factor and Histone ChIP-seq processing pipeline (https://github.com/kundajelab/chipseq_pipeline) following ENCODE specifications. ChIP-seq peaks were called over cell-specific input controls by using MACS2 [73] with a q value of 0.01. Peak calling for each biological replicate was performed separately and reproducible (overlapping) peaks from biological duplicates of each histone modification and BRD4 were averaged and used for downstream data analysis. BEDtools [74] analyses were performed to intersect different sets of genomic regions enriched with examined histone modifications. HOMER [75] was used to annotate ChIP-seq peaks for genomic location and to link these peaks to nearby genes. It was also used for TF binding motif analysis. BigWig files were generated using UCSC bedGraphToBigWig. Genome tracks were generated using the UCSC genome browser (http://genome.ucsc.edu/) with tracks normalized to 1 million reads.

### SE analysis

ChIP-seq peaks of H4K5acK8ac, H3K27ac, and BRD4 (MACS2 q value = 0.01) were subjected to the ROSE algorithm [6, 7] to identify SEs and TEs with default parameters. Enhancers within 12.5-kb of each other were stitched together. All enhancers were ranked by the density of ChIP-seq reads over input. By using the curve of the normalized signals of ChIP-seq within the enhancer region versus the enhancer rank, SEs and TEs were classified as being above or below the point where the tangent to the curve had a slope of 1. BEDtools analyses were performed with SE or TE interval overlaps to obtain the cell type–specific SEs and TEs preferentially enriched with either H4K5acK8ac or H3K27ac. BEDtools coverage was used to obtain the signal at genomic regions by counting ChIP-seq reads over input control within a particular region. The density of reads was calculated as signal divided by the region length (kb). GREAT was used to assign distal regions including SE- or TE-associated genes and their biological functions.

### deepTools analysis

To visualize the ChIP-seq data, *deepTools* modules were used. The computeMatrix was used to calculate the values for heatmaps and summary plots. *Profiler* and *heatmapper* were used to obtain the average profiles of read coverage of the genomes. ChIP-seq bigwig (bw) files were used as score files, and ChIP-seq BED files were used as the genomic regions of interest.

### CAGE transcriptome profiling

RNA was isolated from the nuclear fractions of three biological replicates for each treatment group: DMSO-treated, JQ1-treated, and untreated cells (0316-GSC, U87, and C13NJ). The RNA integrity value of each sample was confirmed by Agilent Bioanalyzer, and then the samples (> 3 μg each) were subjected to CAGE library preparation [45]. At least 15 million reads were sequenced for each. FANTOM CAT V1.0.0 [76] was used to annotate the 5ʹ ends of the CAGE reads. EdgeR V3.18.1 [77] was used to obtain expression values of CAGE libraries as counts per million and to identify differentially expressed genes. For GSEA [49], CAGE transcriptome datasets were analyzed with log_2_ fold change values using edgeR.

### Statistical analysis

Statistical analyses were performed using the R programming language [78] to compute Spearman’s/Pearson’s correlation, test the level of significance for Welch’s *t*-test or Student’s *t*-test, and generate plots with a cut-off of FDR and/or log_2_ fold change values using edgeR (V3.18.1) [77].

### Gene expression analysis

Quantitative reverse-transcription PCR was performed using the QuantStudio 6 real-time PCR system (Applied Biosystems) with the PowerUp SYBR Green PCR Master Mix (Cat. no. A25741, Applied Biosystems), and the primers listed in Supplementary Table 5. Relative gene expression levels were normalized against *β-actin* expression.

### Immunostaining analysis

After fixation for 5 min at room temperature, U87 cells were permeabilized with 1% Triton X-100 in PBS for 20 min at room temperature. Cy3 (GE Healthcare) dye at a 1:4 dye/protein ratio was used to label the H4K5acK8ac antibody. After blocking with Blocking One P (Nacalai Tesque) for 20 min at room temperature, cells were incubated with 2 μg/ml Cy3-conjugated anti-H4K5acK8ac antibody with 2–10 μg/ml Alexa Fluor 488–conjugated histone modification–specific antibody (H3K27ac/CMA309, H3K4me1/ CMA302, or H3K4me3/CMA304) [79] and 1 μg/ml Hoechst 33,342 for 2.5 h at room temperature. Fluorescence images were obtained using a confocal microscope (FV1000; Olympus) with a 60 × UPlanApoN oil immersion lens (numerical aperture, 1.40). Colocalization was analyzed using NIS-Elements Ver4.30 (Nikon).

### RNA interference

For siRNA-mediated knockdown of gene expression, 0316-GSC, U87, and C13NJ cells were transfected with 25 nM siRNA targeting a gene of interest, negative control siRNA (Cat. No. AM4611, ThermoFisher Scientific), or NOTCH1-siRNA (Applied Biosystems) by using Lipofectamine RNAiMAX Transfection Reagent (Cat. No. 13778030, ThermoFisher Scientific). Total RNA was extracted after 72 h of transfection and the knockdown effect was evaluated through gene expression, cell proliferation, and sphere formation analyses.

### CRISPR-Cas9–mediated SE deletion

gRNAs were designed to direct Cas9 to regions flanking H4K5acK8ac- or H3K27ac-preferred SEs (Supplementary Table 5). CRISPR-Cas9–mediated knockout was performed by transfection of a ribonucleoprotein complex consisting of Cas9 (Cat No. 1081058, Integrated DNA Technologies), crRNA (gRNA), and tracrRNA (Cat No. 1075927, Alt-R® CRISPR-Cas9 tracrRNA – ATTO 550, Integrated DNA Technologies) into 0316-GSC cells by using Lipofectamine RNAiMAX Transfection Reagent. For the negative control, a computationally validated Alt-R® CRISPR-Cas9 crRNA (Cat No. 1072544, Integrated DNA Technologies) was used. Twenty-four hours after transfection, to isolate genome-edited cells, cells were subjected to fluorescence-activated cell sorting based on ATTO 550 positivity. Edited cells were screened by genomic PCR to confirm the deletion of the H4K5acK8ac- or H3K27ac-preferred SE regions.

### Cell proliferation assay

Cell proliferation assays were performed using alamarBlue reagent (BUF012A, Bio-Rad). Briefly, cells were seeded at 30,000 cells per well into a 96-well plate containing 100 μl of culture medium per well and then incubated for the indicated period. After the addition of the AlamarBlue reagent, the cells were incubated for a further 4 h, and fluorescence was measured with excitation and emission wavelengths at 535 nm and 600 nm, respectively.

### Sphere formation assay

0316-GSC cells were seeded at 10,000 cells per well into a 96-well plate containing 150 μl of GSC culture medium per well. Fourteen days later, spheres were counted and the sphere formation efficiency (%) was calculated as the number of spheres divided by the number of cells seeded multiplied by 100.

### Supplementary Information

**Additional file 1:** **Figure S1.** Identification of regions that bind H4K5acK8ac preferentially over H3K27ac. **Figure S2.** Effect of JQ1 on genome-wide enrichment of BRD4, H3K27ac, and H4K5acK8ac across glial cell lines. **Figure S3.** Effect of H4K5acK8ac level in regulatory elements on changes in gene expression upon JQ1 treatment in three glial cell lines. **Figure S4.** Effect of JQ1 on specific genes and various biological pathways and molecular functions across the three glial cell lines. **Figure S5.** Defining super-enhancers (SEs) by H4K5acK8ac enrichment ranking in 0316-GSC, U87, and C13NJ cells. **Figure S6.** CRISPR-Cas9–mediated genome editing strategy of H4K5acK8ac-preferred super-enhancers (SEs) in 0316-GSC cells. **Figure S7.** CRISPR-Cas9–mediated genome editing of HOXA7 super-enhancers (SEs) in 0316-GSC cells. **Figure S8.** CRISPR-Cas9–mediated genome editing of H3K27ac-preferred super-enhancers (SEs) in 0316-GSC cells. **Figure S9.** Source data for cropped gel images.

**Additional file 2:** **Table S1.** Pairwise interactions among ChIP-seq peaks across three cell lines (Excel). **Table S2.** H4K5acK8ac- and H3K27ac-preferred promoters and enhancers that were differentially expressed upon JQ1 treatment in 0316-GSC cells (Excel). **Table S3.** Gene set enrichment analysis of genes differentially expressed between JQ1 and DMSO (vehicle control) treatment in three cell lines (Excel). **Table S4.** Cellular and molecular categorization of genes with H4K5acK8ac- or H3K27ac-preferred promoters or SEs in 0316-GSC cells (Excel). **Table S5.** Genes associated with H4K5acK8ac- and H3K27ac-preferred SEs across the three cell lines (Excel). **Table S6.** List of all primers and gRNAs used in this study (Excel).

## Data Availability

All ChIP-seq and CAGE datasets generated in this study have been deposited in the Gene Expression Omnibus (GEO) under accession number GSE178471. The source code used for data analyses and materials are available upon request.
