# Supplementary material for: Defining super-enhancers by highly ranked histone H4 multi-acetylation levels identifies transcription factors associated with glioblastoma stem-like properties
Source: BMC Genomics. 2023 Sep 27;24:574. doi: 10.1186/s12864-023-09659-w (PMC10523799; doi:10.1186/s12864-023-09659-w)
Supplement: Supplementary file 1 — Additional file 1: Figure S1. Identification of regions that bind H4K5acK8ac preferentially over H3K27ac. Figure S2. Effect of JQ1 on genome-wide enrichment of BRD4, H3K27ac, and H4K5acK8ac across glial cell lines. Figure S3. Effect of H4K5acK8ac level in regulatory elements on changes in gene expression upon JQ1 treatment in three glial cell lines. Figure S4. Effect of JQ1 on specific genes and various biological pathways and molecular functions across the three glial cell lines. Figure S5. Defining super-enhancers (SEs) by H4K5acK8ac enrichment ranking in 0316-GSC, U87, and C13NJ cells. Figure S6. CRISPR-Cas9–mediated genome editing strategy of H4K5acK8ac-preferred super-enhancers (SEs) in 0316-GSC cells. Figure S7. CRISPR-Cas9–mediated genome editing of HOXA7 super-enhancers (SEs) in 0316-GSC cells. Figure S8. CRISPR-Cas9–mediated genome editing of H3K27ac-preferred super-enhancers (SEs) in 0316-GSC cells. Figure S9. Source data for cropped gel images. [file 12864_2023_9659_MOESM1_ESM.pdf]

Defining super-enhancers by highly ranked histone H4 multi-acetylation levels identifies transcription factors associated with glioblastoma stem-like properties

Nando D. Das[1], Jen-Chien Chang[2], Chung-Chau Hon[3], S. Thomas Kelly[2], Shinsuke Ito[4], Marina Lizio[3], Bogumil Kaczkowski[5], Hisami Watanabe[1], Keisuke Katsushima[6], Atsushi Natsume[7], Haruhiko Koseki[4,8], Yutaka Kondo[6], Aki Minoda[2,9] and Takashi Umehara[1,10,*]

[1]Laboratory for Epigenetics Drug Discovery, RIKEN Center for Biosystems Dynamics Research, Yokohama, Japan; [2]Laboratory for Cellular Epigenomics, RIKEN Center for Integrative Medical Sciences (IMS), Yokohama, Japan; [3]Laboratory for Genome Information Analysis, RIKEN IMS, Yokohama, Japan; [4]Laboratory of Developmental Genetics, RIKEN IMS, Yokohama, Japan; [5]Laboratory for Applied Regulatory Genomics Network Analysis, RIKEN IMS, Yokohama, Japan; [6]Division of Cancer Biology, Nagoya University Graduate School of Medicine, Nagoya, Japan; [7]Department of Neurosurgery, Nagoya University Graduate School of Medicine, Nagoya, Japan; [8]Immune Regulation, Advanced Research Departments, Graduate School of Medicine, Chiba University, Chiba, Japan; [9]Department of Cell Biology, Faculty of Science, Radboud Institute for Molecular Life Sciences, Radboud University, Nijmegen, Netherlands, [10]PRESTO, Japan Science and Technology Agency, Kawaguchi, Saitama, Japan

[*]Correspondence and requests for materials should be addressed to T.U. (email: takashi.umehara@riken.jp).

Supplementary Figures

Figure S1. Identification of regions that bind H4K5acK8ac preferentially over H3K27ac.

Figure S2. Effect of JQ1 on genome-wide enrichment of BRD4, H3K27ac, and H4K5acK8ac across glial cell lines.

Figure S3. Effect of H4K5acK8ac level in regulatory elements on changes in gene expression upon JQ1 treatment in three glial cell lines.

Figure S4. Effect of JQ1 on specific genes and various biological pathways and molecular functions across the three glial cell lines.

Figure S5. Defining super-enhancers (SEs) by H4K5acK8ac enrichment ranking in 0316-GSC, U87, and C13NJ cells.

Figure S6. CRISPR-Cas9–mediated genome editing strategy of H4K5acK8ac-preferred super-enhancers (SEs) in 0316-GSC cells.

Figure S7. CRISPR-Cas9–mediated genome editing of *HOXA7* super-enhancers (SEs) in 0316-GSC cells.

Figure S8. CRISPR-Cas9–mediated genome editing of H3K27ac-preferred super-enhancers (SEs) in 0316-GSC cells.

Figure S9. Source data for cropped gel images.

Figure S1

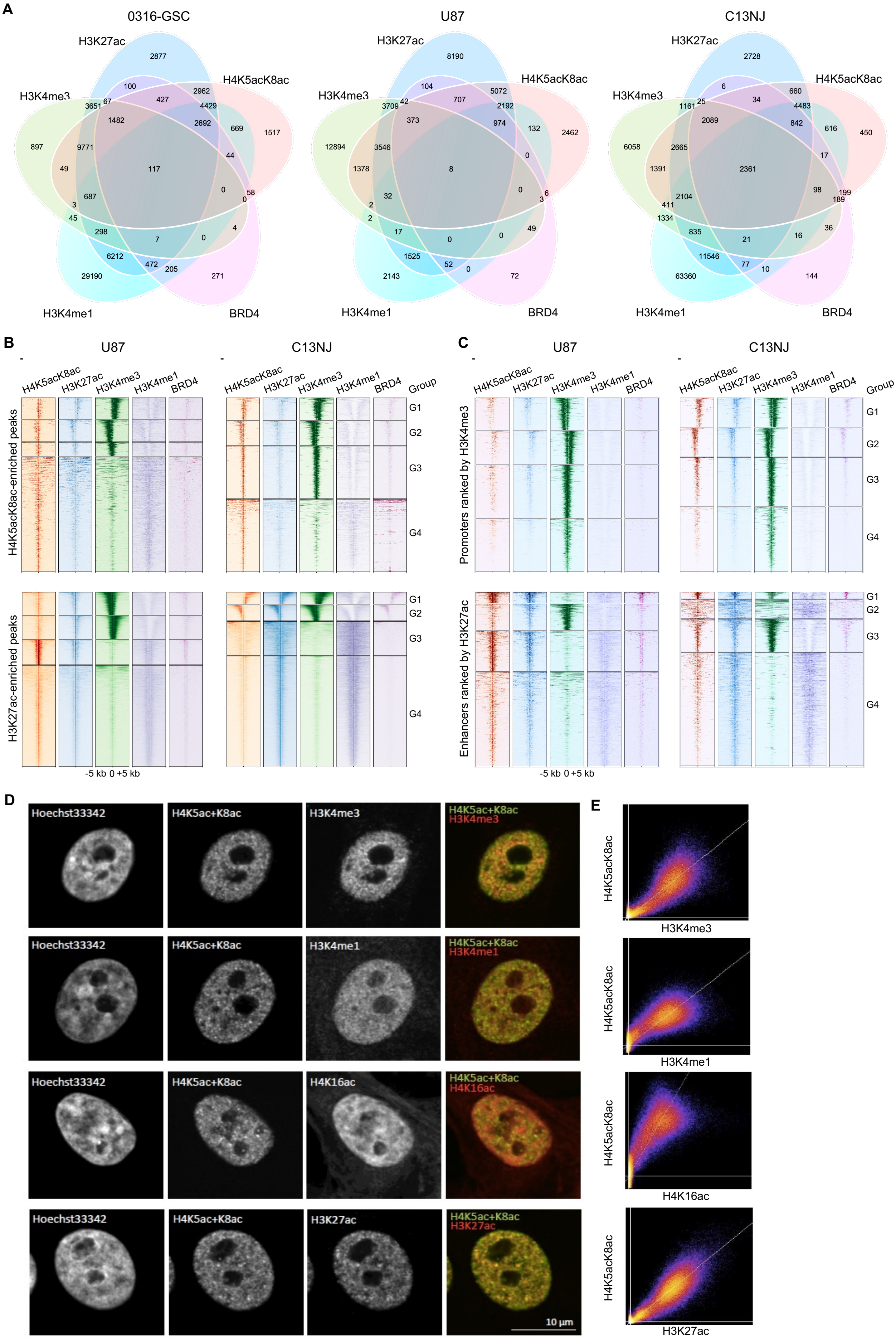
A
0316-GSC
U87
C13NJ
H3K27ac
H4K5acK8ac
H3K4me3
H3K4me1
BRD4
B
U87
C13NJ
H4K5acK8ac
H3K27ac
H3K4me3
H3K4me1
BRD4
Group
G1
G2
G3
G4
H4K5acK8ac-enriched peaks
H3K27ac-enriched peaks
-5 kb 0 +5 kb
C
Promoters ranked by H3K4me3
Enhancers ranked by H3K27ac
D
Hoechst33342
H4K5ac+K8ac
H3K4me3
H3K4me1
H4K16ac
H3K27ac
10 µm
E
H4K5acK8ac
H3K4me3
H3K4me1
H4K16ac
H3K27ac

Figure S1 (continued)

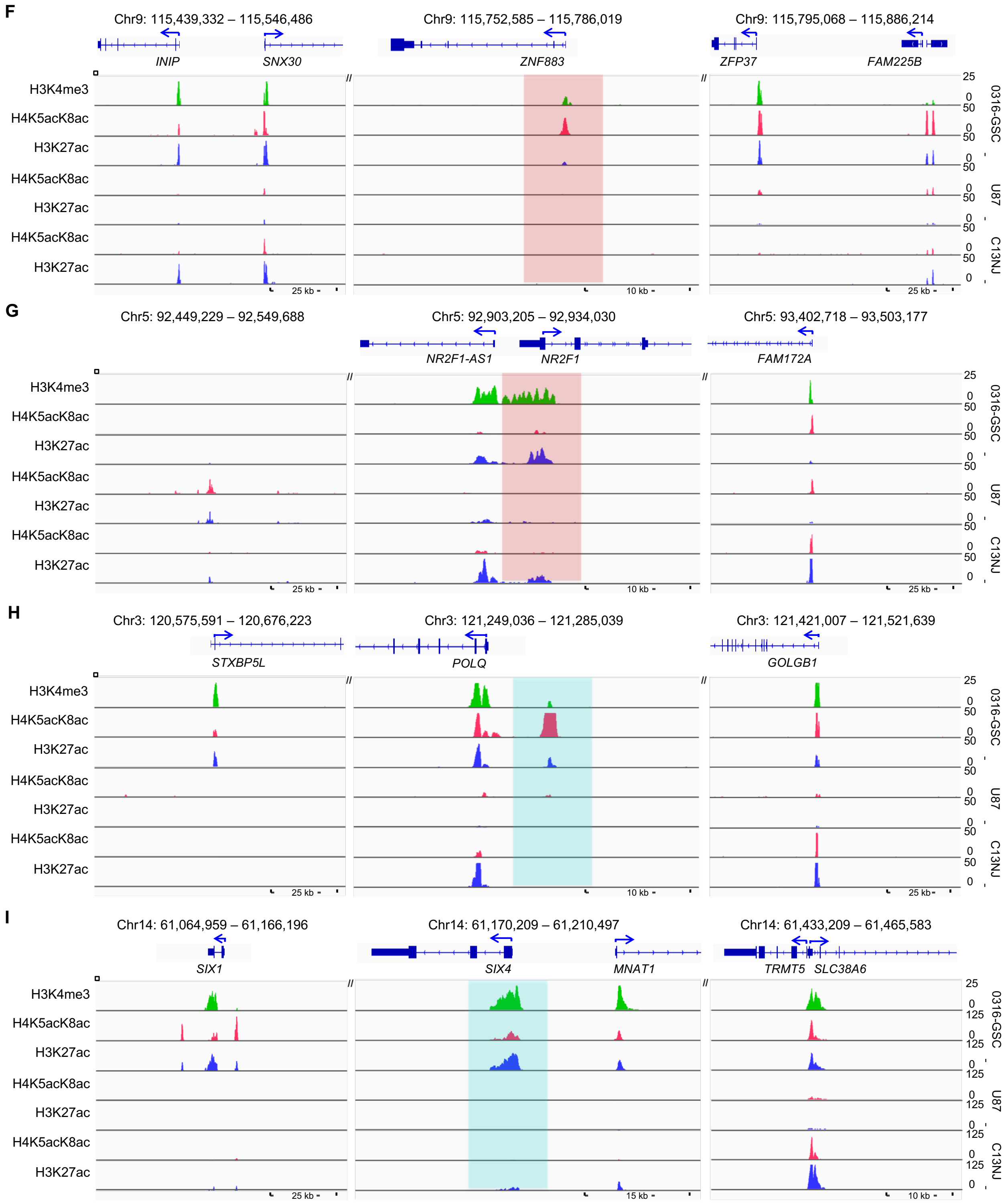

Figure S1. Identification of regions that bind H4K5acK8ac preferentially over H3K27ac. A Venn diagrams representing the overlap of ChIP-seq peaks of H4K5acK8ac with H3K27ac, H3K4me1, H3K4me3, and BRD4 across 0316-GSC, U87, and C13NJ cell lines. **B** and **C** Heatmaps representing different ChIP-seq datasets (H4K5acK8ac, H3K27ac, H3K4me3, H3K4me1, and BRD4) in U87 and C13NJ cell lines. Data are from within ± 5-kb from the summit of H4K5acK8ac-enriched peaks (upper, **B**) and H3K27ac-enriched peaks (lower, B), and promoters defined by H3K4me3 at the promoters (upper, **C**) and enhancers defined by H3K27ac located outside a promoter (lower, **C**). Each row represents a single peak. Color density indicates the average enrichment of each mark at the selected regions. H4K5acK8ac- or H3K27ac-enriched peaks (**B**) and promoters or enhancers (**C**) are each clustered into four groups (G1 to G4) according to the ChIP-seq profiles. **D** Immunostaining analysis demonstrating the colocalization of H4K5acK8ac with H3K4me3, H3K4me1, H4K16ac, and H3K27ac marks in U87 cells. Hoechst 33342 was used for nuclear staining. Scale bar, 10 µm. **E** Correlation of immunofluorescence signal between H4K5acK8ac and other specific marks in U87 cells. **F–I** Representative 0316-GSC-specific ChIP-seq tracks of H4K5acK8ac-preferred ($\log_2$ fold-change (FC) [H4K5acK8ac/H3K27ac] > 1) and H3K27ac-preferred ($\log_2$FC [H4K5acK8ac/H3K27ac] < −1) promoters (**F** and **G**) and enhancers (**H** and **I**).

Figure S2

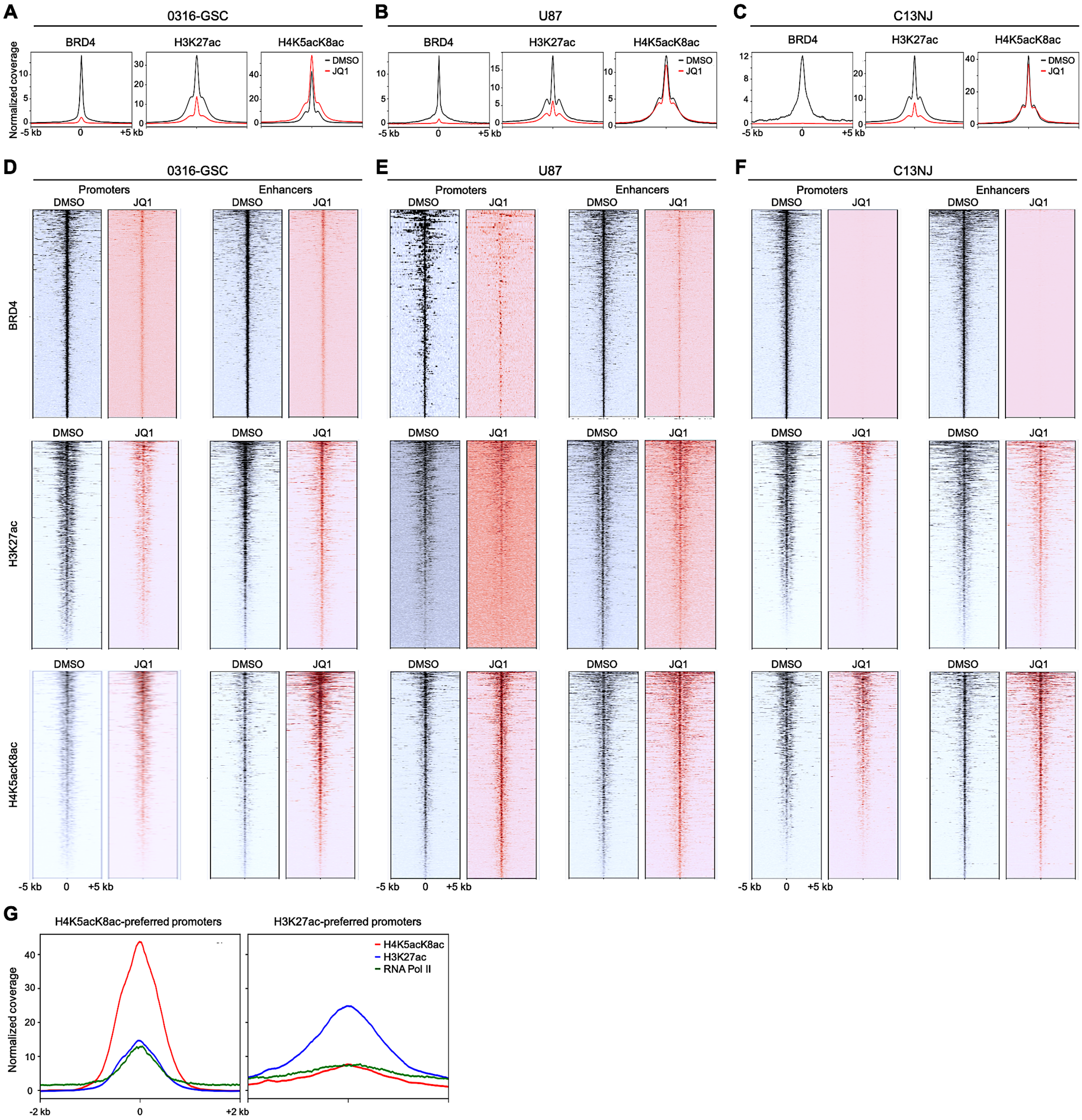

Figure S2. Effect of JQ1 on genome-wide enrichment of BRD4, H3K27ac, and H4K5acK8ac across glial cell lines. **A**–**C** ChIP-seq meta-profiles for dimethyl sulfoxide (DMSO; vehicle control) and JQ1-treated cells showing the average read counts (reads per million; RPM) of ± 5 kb regions from the summit of BRD4-, H3K27ac-, and H4K5acK8ac-enrichment. Compared with treatment with DMSO, treatment with 5 μM JQ1 for 24 h led to a global loss of BRD4 and H3K27ac across the three cell lines, and the enrichment of H4K5acK8ac in 0316-GSC but not U87 or C13NJ cells. **D**–**F** Heatmaps of ChIP-seq datasets of BRD4, H3K27ac, and H4K5acK8ac upon JQ1 treatment in 0316-GSC cells (**D**), U87 (**E**), and C13NJ (**F**) cells within ± 5 kb from the summit of active promoters (defined by cell type–specific H3K4me3) (left) or active enhancers (defined by cell type–specific H3K27ac located outside promoter) (right). Each row represents a single peak. Color density indicates the average enrichment of each mark at promoters and enhancers. **G** Colocalization of RNAP II with promoters. ChIP-seq meta-profiles show the association of RNAP II (green) with H4K5acK8ac-preferred (red) and H3K27ac-preferred (blue) promoters.

Figure S3

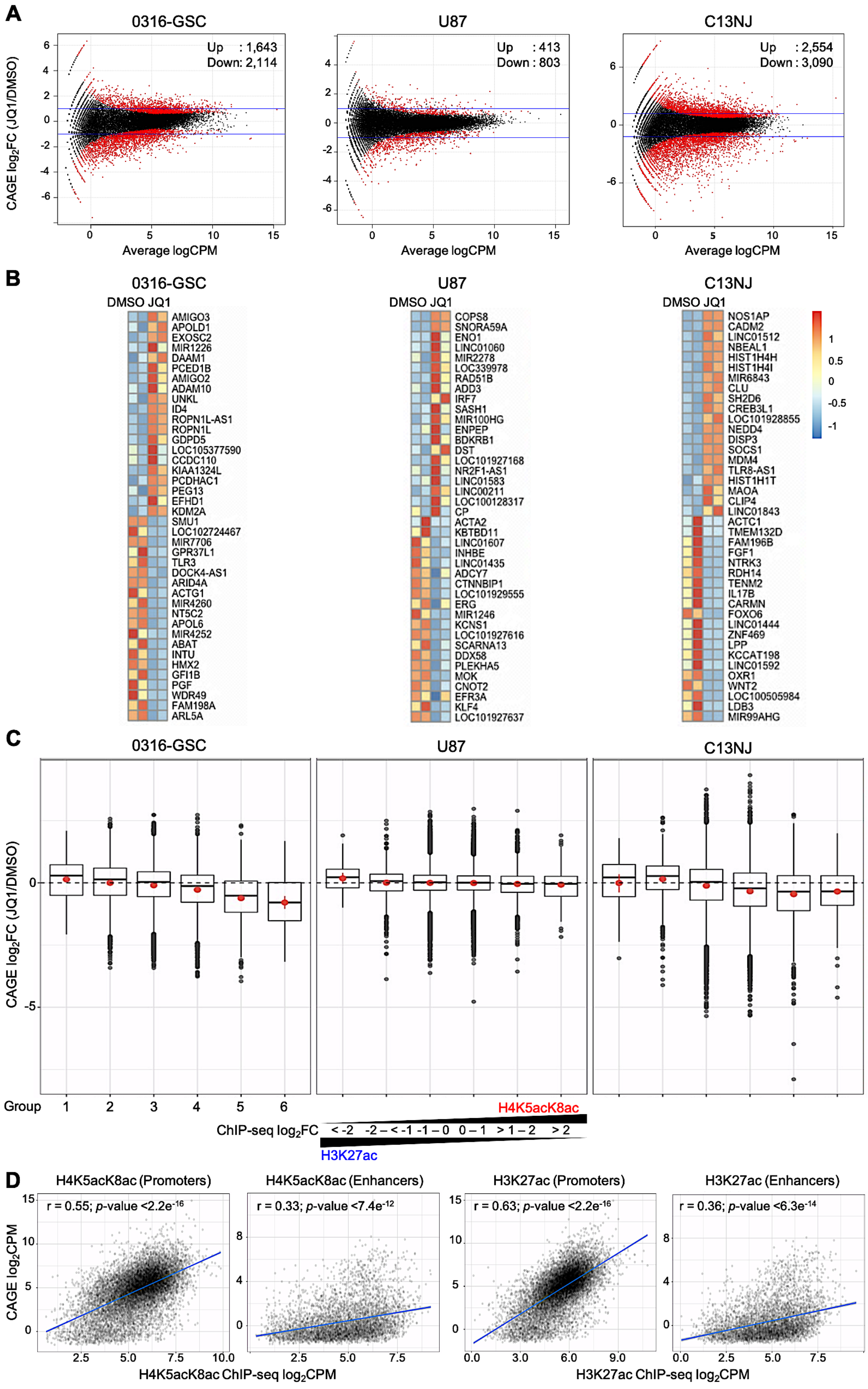
A
0316-GSC
Up : 1,643
Down: 2,114
U87
Up : 413
Down : 803
C13NJ
Up : 2,554
Down : 3,090
CAGE log2FC (JQ1/DMSO)
Average logCPM
B
0316-GSC
DMSO JQ1
AMIGO3 APOLD1 EXOSC2 MIR1226 DAAM1 PCED1B AMIGO2 ADAM10 UNKL ID4 ROPN1L-AS1 ROPN1L GDPD5 LOC105377590 CCDC110 KIAA1324L PCDHAC1 PEG13 EFHD1 KDM2A SMU1 LOC102724467 MIR7706 GPR37L1 TLR3 DOCK4-AS1 ARID4A ACTG1 MIR4260 NT5C2 APOL6 MIR4252 ABAT INTU HMX2 GFI1B PGF WDR49 FAM198A ARL5A
U87
DMSO JQ1
COPS8 SNORA59A ENO1 LINC01060 MIR2278 LOC339978 RAD51B ADD3 IRF7 SASH1 MIR100HG ENPEP BDKRB1 DST LOC101927168 NR2F1-AS1 LINC01583 LINC00211 LOC100128317 CP ACTA2 KBTBD11 LINC01607 INHBE LINC01435 ADCY7 CTNNBIP1 LOC101929555 ERG MIR1246 KCNS1 LOC101927616 SCARNA13 DDX58 PLEKHA5 MOK CNOT2 EFR3A KLF4 LOC101927637
C13NJ
DMSO JQ1
NOS1AP CADM2 LINC01512 NBEAL1 HIST1H4H HIST1H4I MIR6843 CLU SH2D6 CREB3L1 LOC101928855 NEDD4 DISP3 SOCS1 MDM4 TLR8-AS1 HIST1H1T MAOA CLIP4 LINC01843 ACTC1 TMEM132D FAM196B FGF1 NTRK3 RDH14 TENM2 IL17B CARMN FOXO6 LINC01444 ZNF469 LPP KCCAT198 LINC01592 OXR1 WNT2 LOC100505984 LDB3 MIR99AHG
1 0.5 0 -0.5 -1
C
0316-GSC
U87
C13NJ
CAGE log2FC (JQ1/DMSO)
Group 1 2 3 4 5 6
H4K5acK8ac
ChIP-seq log2FC < -2 -2 – < -1 -1 – 0 0 – 1 > 1 – 2 > 2
H3K27ac
D
H4K5acK8ac (Promoters)
r = 0.55; p-value <2.2e-16
H4K5acK8ac (Enhancers)
r = 0.33; p-value <7.4e-12
H3K27ac (Promoters)
r = 0.63; p-value <2.2e-16
H3K27ac (Enhancers)
r = 0.36; p-value <6.3e-14
CAGE log2CPM
H4K5acK8ac ChIP-seq log2CPM
H3K27ac ChIP-seq log2CPM

Figure S3. Effect of H4K5acK8ac level in regulatory elements on changes in gene expression upon JQ1 treatment in three glial cell lines. **A** Relative expression of genes upon JQ1 treatment. Numbers of genes significantly upregulated or downregulated by 24-h treatment with 5 µM JQ1 relative to DMSO (false discovery rate < 0.05; CAGE $\log_2$FC [JQ1/DMSO] > 0.5 or < −0.5, respectively) are shown for each of the three cell lines. **B** Heatmaps of the top 20 genes differentially upregulated and top 20 genes differentially downregulated by JQ1 are shown based on the ranking of CAGE $\log_2$FC in each cell line. **C** Box plots of relative expression of genes upon JQ1 treatment for promoters or enhancers grouped by their level of enrichment of H4K5acK8ac over H3K27ac. Groups 1–3 are defined by ChIP-seq $\log_2$FC (H4K5acK8ac/H3K27ac) of < −2, −2 to < −1, and −1 to 0, respectively (H3K27ac-preferred; $\log_2$FC < −1). Groups 4–6 are defined by ChIP-seq $\log_2$FC 0 to 1, >1 to 2, and >2, respectively (H4K5acK8ac-preferred; $\log_2$FC > 1). **D** Scatter plots of the change in gene expression upon JQ1 treatment (CAGE $\log_2$FC) versus levels of histone modifications (ChIP-seq $\log_2$CPM) for genes associated with H4K5acK8ac-preferred and H3K27ac-preferred promoters or enhancers in 0316-GSC cells. The blue line indicates the linear fit; *r* indicates Spearman's correlation (n = 2 biological replicates for each histone modification and FC value of each gene is the average of the FCs of the two biological replicates).

Figure S4

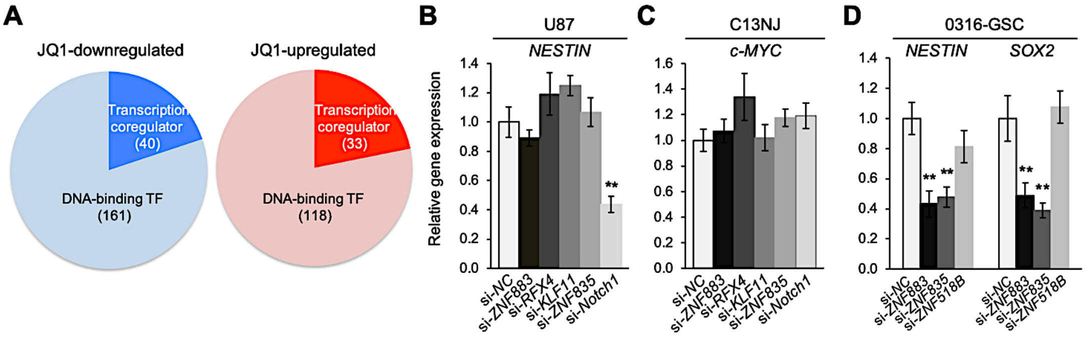

E

Categories	H4K5acK8ac	H3K27ac
JQ1-downregulated ($log_2FC \leq -0.5$)	*ZNF883, RFX4, KLF11, ZNF835, SMARCD3, STAT5A, HNF4G, IFI16, NFATC4, TWIST2, METAP1D, FOXI3, NMI, ZBTB20, NEUROD1, ETV7*	*E2F8, ETV4, E2F3, ZBTB16, PPARGC1A, MYCL, NR2F1, TNFRSF19, ZEB1, MUM1*
JQ1-upregulated ($log_2FC \geq 0.5$)	*ZNF280C, PRDM2, PEPD*	*SOX4, EGR2, SERTAD1, TAF10, EGR1, JUNB, SMAD7, STAT3, MXD1, PHC2, ID2, MAFF, TBX2, DLX1, MYCBP, EPAS1, ZIC2, ZIC4, IRF2BPL, NPAS1, TEAD1, MAFG*

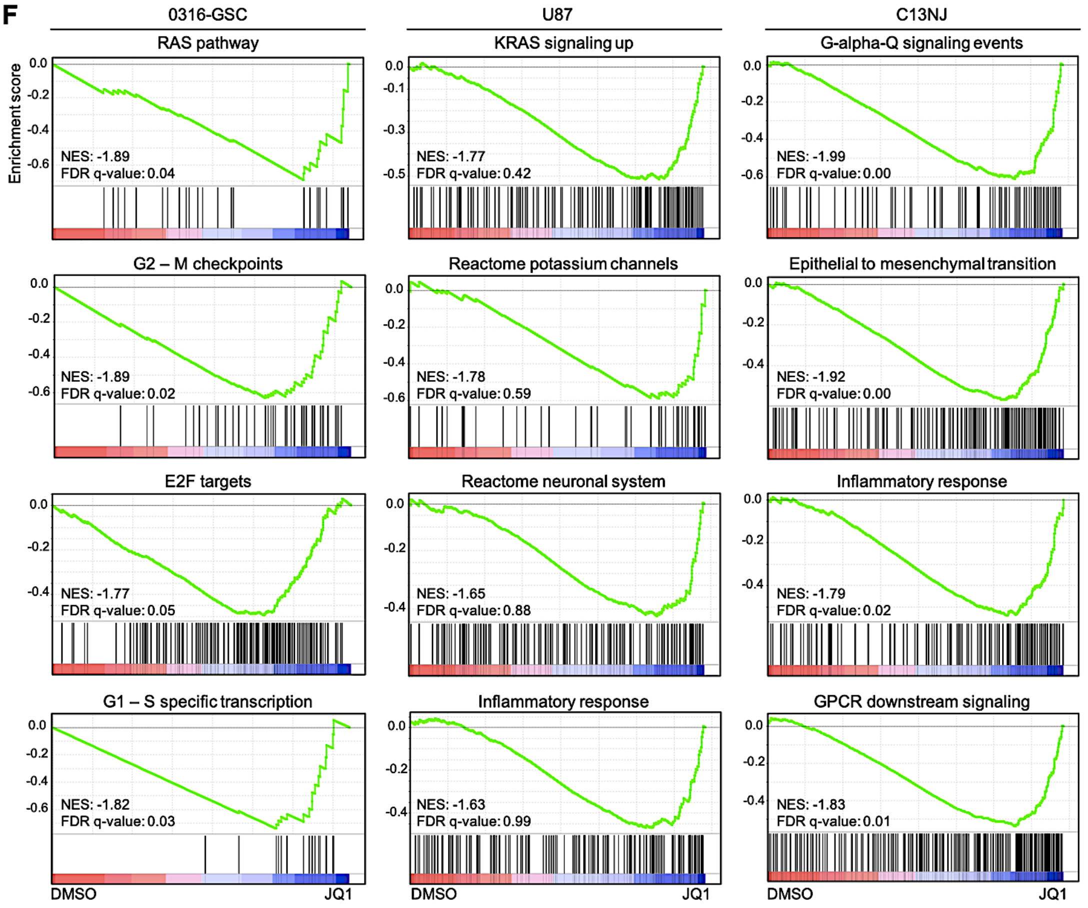

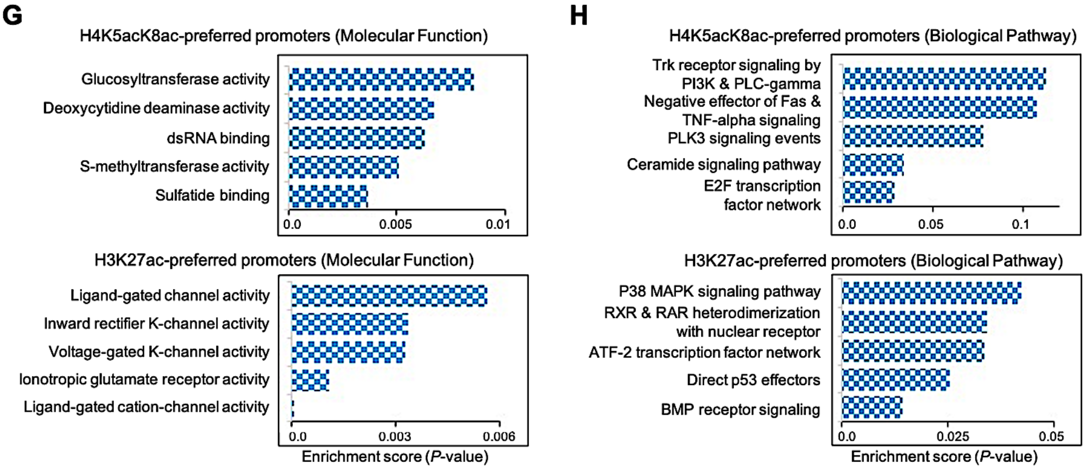

Figure S4. Effect of JQ1 on specific genes and various biological pathways and molecular functions across the three glial cell lines. A JQ1-downregulated and upregulated genes involved in transcriptional activity were categorized as transcription coregulators or DNA-binding transcription factors (TFs). **B** and **C** Unlike siRNA knockdown of *NOTCH1*, siRNA knockdown of TF candidate genes with H4K5acK8ac-preferred promoters did not result in significant changes in expression of the stem cell marker gene *NESTIN* in U87 (**B**) cells. Neither siRNA knockdown of *NOTCH1* nor siRNA knockdown of the set of TF candidate genes with H4K5acK8ac-preferred promoters resulted in significant changes in c-*Myc* expression in C13NJ (**C**) cells (n = 3). **D** Comparative siRNA knockdown of selected JQ1-unaffected and JQ1-downregulated genes encoding zinc-finger TFs with H4K5acK8ac-preferred promoters in 0316-GSC. siRNA knockdown of the JQ1-unaffected gene, *ZNF518B*, did not suppress the expression of stem cell marker genes *NESTIN* and *SOX2* (n = 3), but siRNA knockdown of JQ1-downregulated genes, *ZNF833* and *ZNF835*, did significantly suppress the expression of these genes. Data are means ± SEM. **, $P < 0.01$ (two-tailed Student's *t*-test). **E** JQ1-downregulated and upregulated TF candidate genes are shown categorized according to whether they have H4K5acK8ac- or H3K27ac-preferred promoters in 0316-GSC cells. **F** GSEA results of gene ontology (GO) biological pathways significantly over-represented in the sets of JQ1-downregulated genes in 0316-GSC, U87, and C13NJ are shown (NES; Normalized Enrichment Score). **G** and **H** GO molecular functions (**G**) and biological pathways (**H**) that are significantly over-represented in JQ1-downregulated genes associated with H4K5acK8ac- (upper) or H3K27ac-preferred (lower) promoters in 0316-GSC cells are shown.

Figure S5

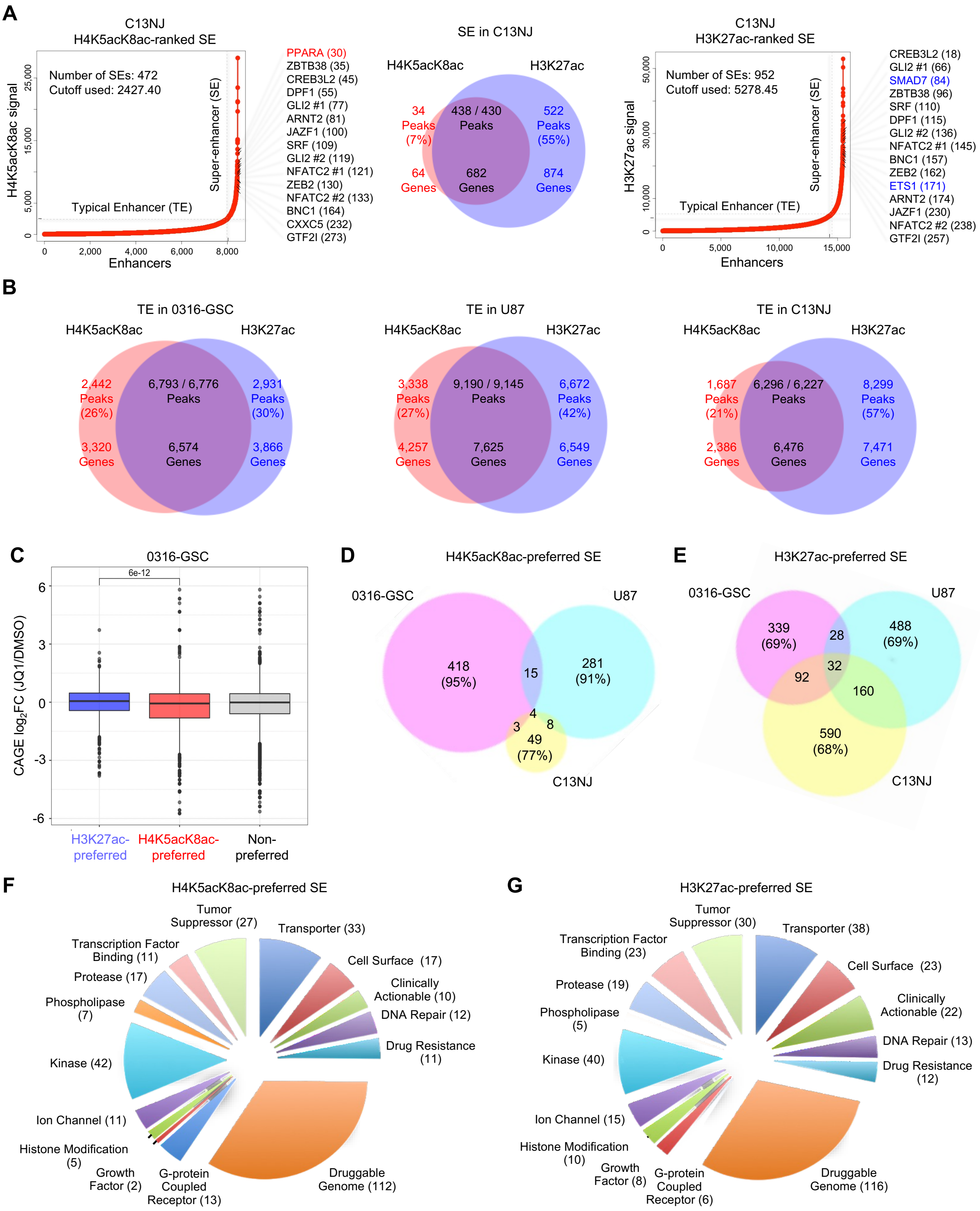
A
C13NJ
H4K5acK8ac-ranked SE
Number of SEs: 472
Cutoff used: 2427.40
Super-enhancer (SE)
Typical Enhancer (TE)
H4K5acK8ac signal
Enhancers
PPARA (30)
ZBTB38 (35)
CREB3L2 (45)
DPF1 (55)
GLI2 #1 (77)
ARNT2 (81)
JAZF1 (100)
SRF (109)
GLI2 #2 (119)
NFATC2 #1 (121)
ZEB2 (130)
NFATC2 #2 (133)
BNC1 (164)
CXXC5 (232)
GTF2I (273)
SE in C13NJ
H4K5acK8ac
H3K27ac
34 Peaks (7%)
438 / 430 Peaks
522 Peaks (55%)
64 Genes
682 Genes
874 Genes
C13NJ
H3K27ac-ranked SE
Number of SEs: 952
Cutoff used: 5278.45
Super-enhancer (SE)
Typical Enhancer (TE)
H3K27ac signal
Enhancers
CREB3L2 (18)
GLI2 #1 (66)
SMAD7 (84)
ZBTB38 (96)
SRF (110)
DPF1 (115)
GLI2 #2 (136)
NFATC2 #1 (145)
BNC1 (157)
ZEB2 (162)
ETS1 (171)
ARNT2 (174)
JAZF1 (230)
NFATC2 #2 (238)
GTF2I (257)
B
TE in 0316-GSC
H4K5acK8ac
H3K27ac
2,442 Peaks (26%)
6,793 / 6,776 Peaks
2,931 Peaks (30%)
3,320 Genes
6,574 Genes
3,866 Genes
TE in U87
H4K5acK8ac
H3K27ac
3,338 Peaks (27%)
9,190 / 9,145 Peaks
6,672 Peaks (42%)
4,257 Genes
7,625 Genes
6,549 Genes
TE in C13NJ
H4K5acK8ac
H3K27ac
1,687 Peaks (21%)
6,296 / 6,227 Peaks
8,299 Peaks (57%)
2,386 Genes
6,476 Genes
7,471 Genes
C
0316-GSC
6e-12
CAGE log_2FC (JQ1/DMSO)
H3K27ac-preferred
H4K5acK8ac-preferred
Non-preferred
D
H4K5acK8ac-preferred SE
0316-GSC
U87
418 (95%)
15
281 (91%)
4
3
8
49 (77%)
C13NJ
E
H3K27ac-preferred SE
0316-GSC
U87
339 (69%)
28
488 (69%)
32
92
160
590 (68%)
C13NJ
F
H4K5acK8ac-preferred SE
Tumor Suppressor (27)
Transporter (33)
Transcription Factor Binding (11)
Cell Surface (17)
Protease (17)
Clinically Actionable (10)
Phospholipase (7)
DNA Repair (12)
Drug Resistance (11)
Kinase (42)
Ion Channel (11)
Histone Modification (5)
Growth Factor (2)
G-protein Coupled Receptor (13)
Druggable Genome (112)
G
H3K27ac-preferred SE
Tumor Suppressor (30)
Transporter (38)
Transcription Factor Binding (23)
Cell Surface (23)
Protease (19)
Clinically Actionable (22)
Phospholipase (5)
DNA Repair (13)
Kinase (40)
Drug Resistance (12)
Ion Channel (15)
Histone Modification (10)
Growth Factor (8)
G-protein Coupled Receptor (6)
Druggable Genome (116)

Figure S5 (continued)

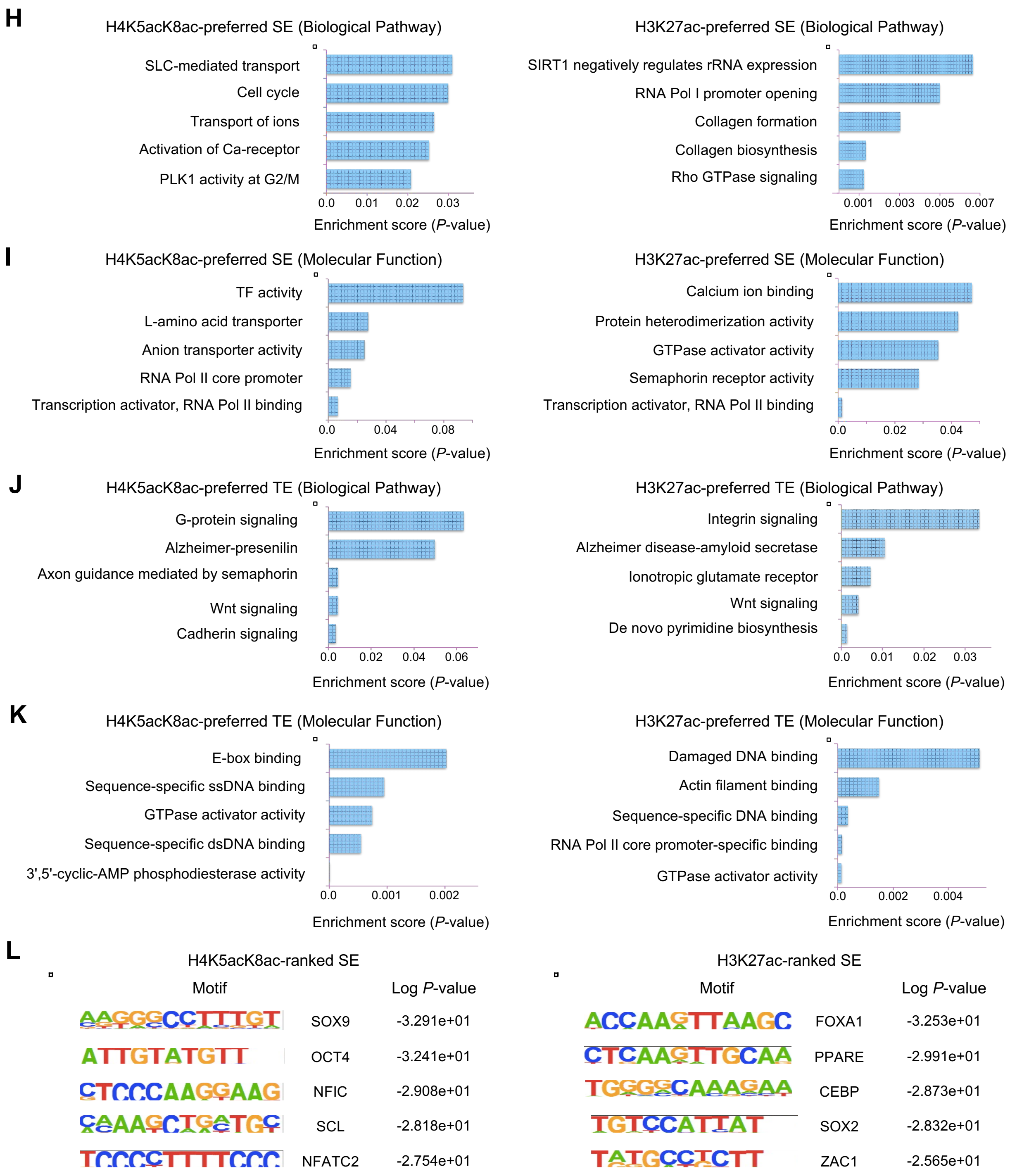

Figure S5. Defining super-enhancers (SEs) by H4K5acK8ac enrichment ranking in 0316-GSC, U87, and C13NJ cells. A Enhancers in C13NJ cells were ranked by H4K5acK8ac (left) or H3K27ac (right) signal level by using the ROSE algorithm; those with extremely high signals were defined as SEs (see Methods in the main text for threshold calculations). JQ1-downregulated (*i.e.*, CAGE $\log_2$FC < −0.5) transcription factor (TF) candidate genes with H4K5acK8ac- (red) or H3K27ac-preferred (blue) SEs are shown. Venn diagrams (middle) show the number of peaks and associated genes with H4K5acK8ac- or H3K27ac-preferred SEs or both (the intersection, purple). **B** Venn diagrams of genes with H4K5acK8ac- and/or H3K27ac-preferred typical enhancers (TEs) across three cell lines. **C** Box plots of change in gene expression levels upon JQ1 treatment in 0316-GSC. Genes associated with enhancers (peaks > 1-kb from the TSS) are grouped by the enrichment level of H4K5acK8ac over H3K27ac. The H3K27ac-, H4K5acK8ac-, and non-preferred peaks associated with enhancers are defined by ChIP-seq $\log_2$FC (H4K5acK8ac/H3K27ac): $\log_2$FC < −1, $\log_2$FC > 1, and $-1 \le \log_2$FC ≤ 1 (n = 2 biological replicates for each histone modification and FC value of each gene is the average of the FCs of the two biological replicates). **D** and **E** Venn diagrams showing cell-type specificities of genes with H4K5acK8ac-preferred (**D**) and H3K27ac-preferred (**E**) SEs. **F** and **G** Pie charts of genes with H4K5acK8ac-preferred (**F**) and H3K27ac-preferred (**G**) SEs in 0316-GSC are shown; the genes were identified by integrating the SE-associated genes with information in the Drug-Gene Interaction Database. **H**–**K** The indicated gene ontology (GO) molecular function and biological pathway categories were over-represented in JQ1-downregulated genes associated with H4K5acK8ac-preferred (left) or H3K27ac-preferred (right) SEs and TEs (**H** and **I**: SEs; **J** and **K**: TEs) in 0316-GSC cells. **L** TF-binding motifs within the H4K5acK8ac (left)- and H3K27ac (right)-ranked SEs. The motifs were found *de novo* by HOMER and then linked to the best matching known motifs. The occurrence panels represent the enrichment of the motif within 500 bp of H4K5acK8ac- and H3K27ac-ranked SEs.

Figure S6

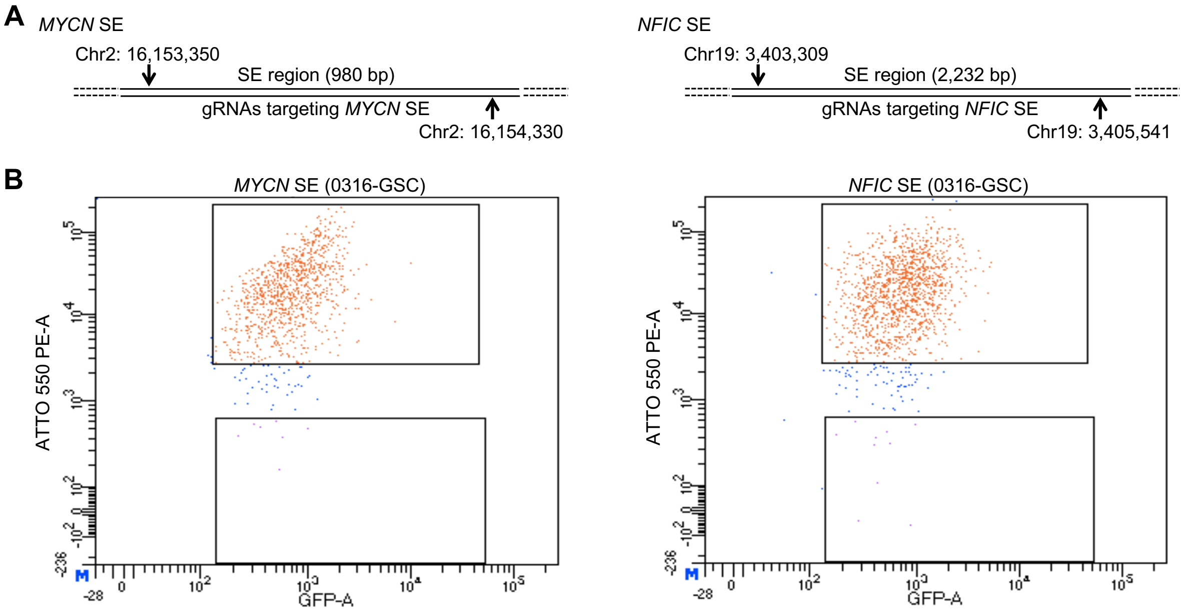

Figure S6. CRISPR-Cas9–mediated genome editing strategy of H4K5acK8ac-preferred super-enhancers (SEs) in 0316-GSC cells. A CRISPR-Cas9 knockout strategy for the SEs associated with *MYCN* and *NFIC* in 0316-GSC cells. Guide RNAs (gRNAs; vertical arrows) show the position for deletion of each SE. **B** Fluorescence-activated cell sorting of the SE-edited cells. CRISPR-Cas9–mediated knockout was performed by transfection of a ribonucleoprotein complex consisting of Cas9, crRNA (gRNA), and tracrRNA conjugated with ATTO 550. The sorted ATTO 550 positive cells (upper box) were subjected for further analysis.

Figure S7

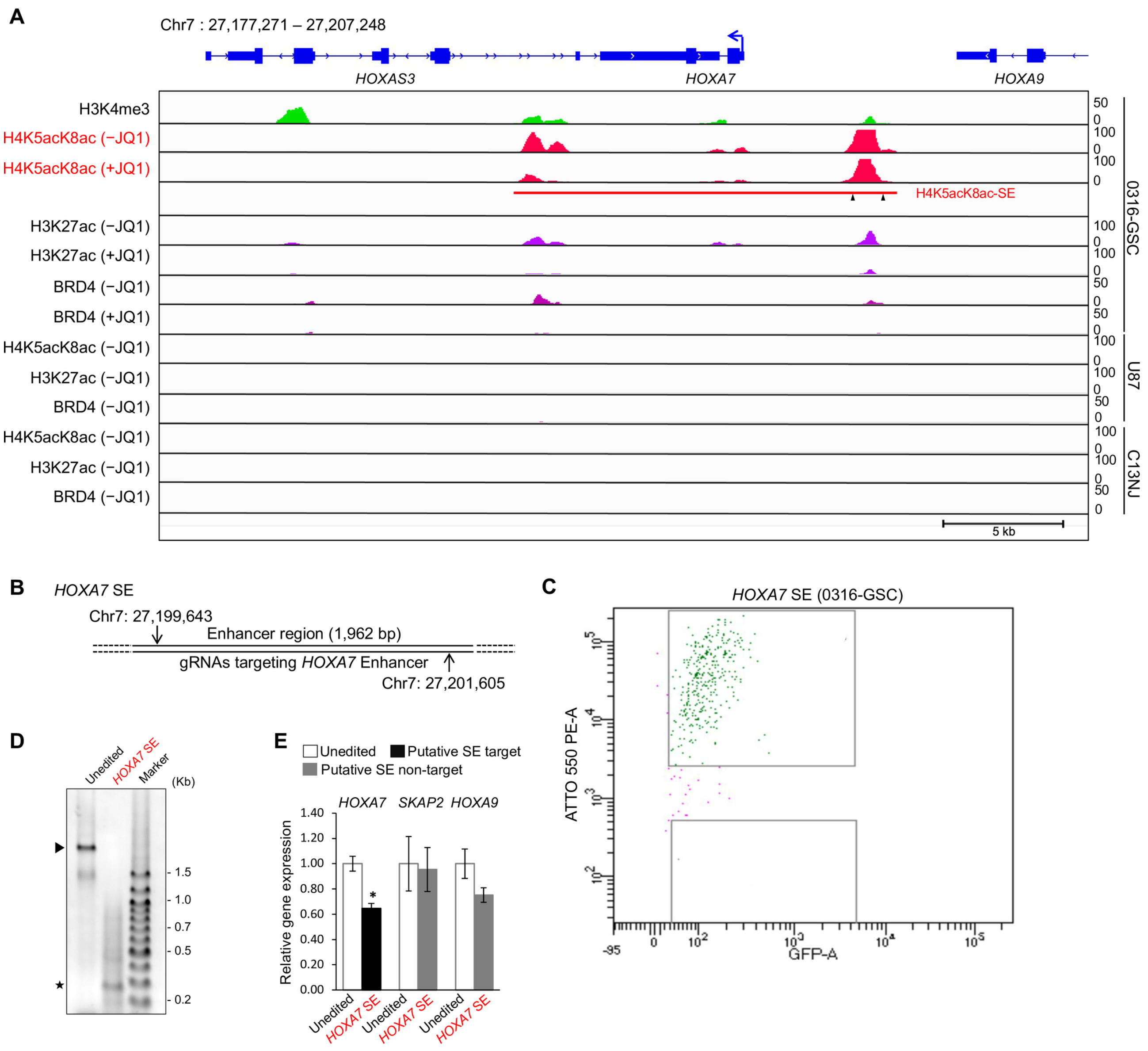

Figure S7. CRISPR-Cas9–mediated genome editing of *HOXA7* SE in 0316-GSC cells. A Representative ChIP-seq occupancy tracks of genes with H4K5acK8ac-preferred SE associated with *HOXA7*. The arrowheads under the SE bar show the position for CRISPR-Cas9–mediated deletion of the SE region. ChIP-seq reads were averaged from two biological replicates. **B** CRISPR-Cas9 knockout strategy. Guide RNAs (gRNAs; vertical arrows) show the position for deletion of each enhancer in SE. **C** Fluorescence-activated cell sorting of the SE-edited cells. CRISPR-Cas9–mediated knockout was performed by transfection of a ribonucleoprotein complex consisting of Cas9, crRNA (gRNA), and tracrRNA conjugated with ATTO 550. The sorted ATTO 550 positive cells (upper box) were subjected for further analysis. **D** Expected band sizes of genomic DNA for unedited (arrowhead) and enhancer-edited (asterisk) samples: the results show the deletion of the *HOXA7* SE. Image of the uncropped gel is shown in Figure S9B. **E** RT-qPCR analysis of the expression of putative target TF genes associated with SE and non-target genes (n= 3). Data are means ± SEM. *, $P < 0.05$ (two-tailed Student's *t*-test).

Figure S8

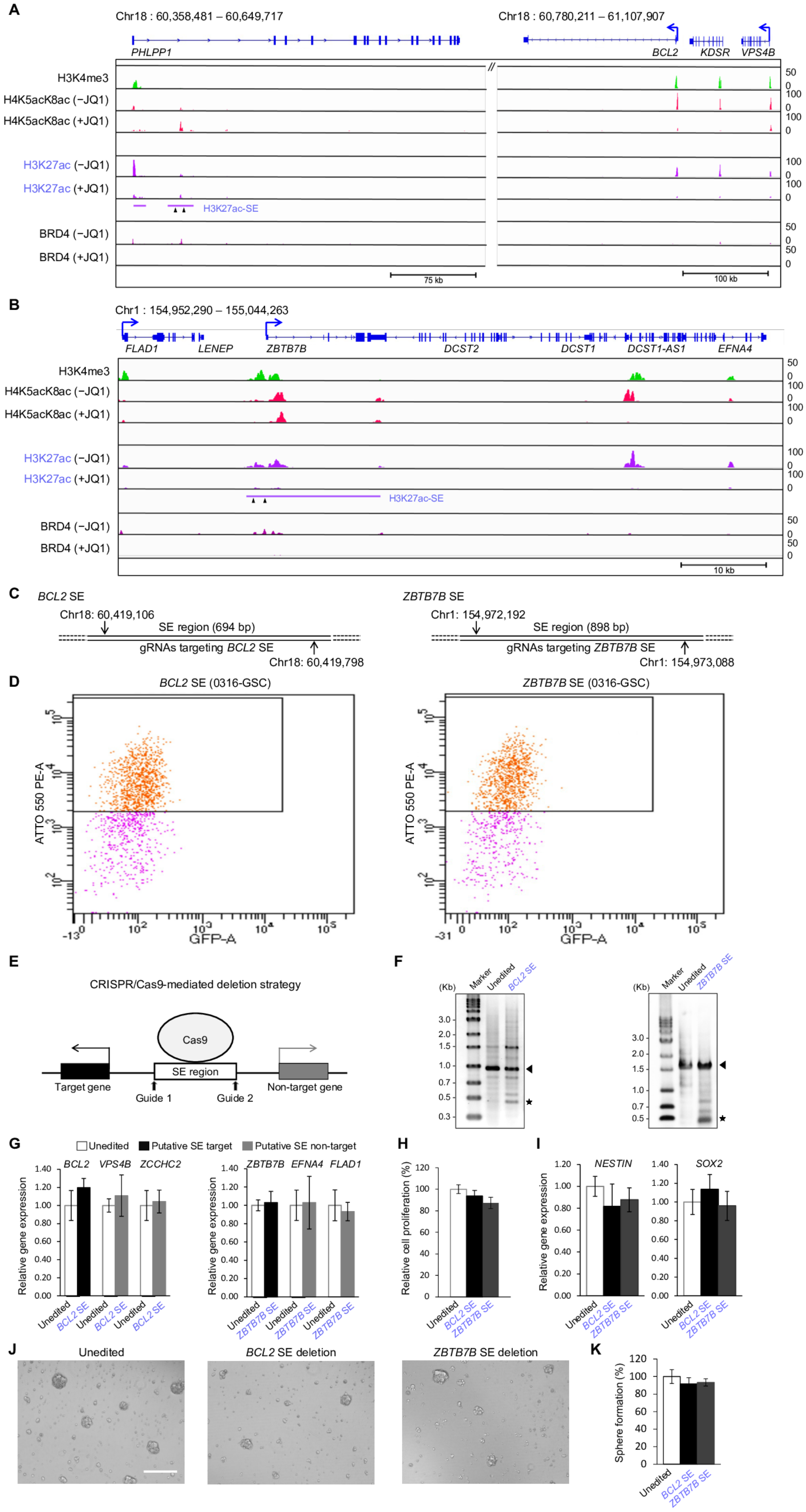
A
Chr18 : 60,358,481 – 60,649,717
Chr18 : 60,780,211 – 61,107,907
PHLPP1
BCL2
KDSR
VPS4B
H3K4me3
H4K5acK8ac (−JQ1)
H4K5acK8ac (+JQ1)
H3K27ac (−JQ1)
H3K27ac (+JQ1)
H3K27ac-SE
BRD4 (−JQ1)
BRD4 (+JQ1)
75 kb
100 kb
B
Chr1 : 154,952,290 – 155,044,263
FLAD1
LENEP
ZBTB7B
DCST2
DCST1
DCST1-AS1
EFNA4
10 kb
C
BCL2 SE
Chr18: 60,419,106
SE region (694 bp)
gRNAs targeting BCL2 SE
Chr18: 60,419,798
ZBTB7B SE
Chr1: 154,972,192
SE region (898 bp)
gRNAs targeting ZBTB7B SE
Chr1: 154,973,088
D
BCL2 SE (0316-GSC)
ZBTB7B SE (0316-GSC)
ATTO 550 PE-A
GFP-A
E
CRISPR/Cas9-mediated deletion strategy
Cas9
SE region
Target gene
Guide 1
Guide 2
Non-target gene
F
(Kb)
Marker
Unedited
BCL2 SE
ZBTB7B SE
G
Unedited
Putative SE target
Putative SE non-target
BCL2
VPS4B
ZCCHC2
ZBTB7B
EFNA4
FLAD1
Relative gene expression
H
Relative cell proliferation (%)
I
NESTIN
SOX2
J
Unedited
BCL2 SE deletion
ZBTB7B SE deletion
K
Sphere formation (%)

Figure S8. CRISPR-Cas9–mediated genome editing of H3K27ac-preferred super-enhancers (SEs) in 0316-GSC cells. **A** ChIP-seq occupancy tracks of H3K27ac-preferred SEs of *BCL2*. **B** ChIP-seq occupancy tracks of H3K27ac-preferred SEs of *ZBTB7B*. Arrowheads under the SE bar show the position for CRISPR-Cas9–mediated deletion of the SE region. ChIP-seq reads were averaged from two biological replicates. **C** CRISPR-Cas9 knockout strategy. Guide RNAs (gRNAs; vertical arrows) show the position for deletion of each SE. **D** Fluorescence-activated cell sorting of the SE-edited cells. CRISPR-Cas9–mediated knockout was performed by transfection of a ribonucleoprotein complex consisting of Cas9, crRNA (gRNA), and tracrRNA conjugated with ATTO 550. The sorted ATTO 550 positive cells (upper box) were subjected for further analysis. **E** Schematic representation showing the CRISPR-Cas9–mediated genome editing approach for SEs. Guide indicates gRNA. **F** Expected band sizes of genomic DNA for unedited (arrowhead) and SE-edited (asterisk) samples: the results show the deletion of the SE associated with *BCL2* and *ZBTB7B*. Images of the uncropped gel are shown in Figure S9C. **G** RT-qPCR analysis of the expression of putative target TF genes associated with enhancers (En) in SEs and non-target genes (n= 3). **H** Cell proliferation rates at 4 days after SE deletion (n = 4). **I** Expression of stem cell marker genes, *NESTIN* (left) and *SOX2* (right), at 4 days after SE deletion (n = 3). **J** Phase-contrast images of 0316-GSC cells at 14 days after SE deletion. Images are representative of three independent experiments. Scale bar, 50 µm. **K** *In vitro* sphere formation efficiency of 0316-GSC cells at 2 weeks after SE deletion (n = 3).

Figure S9

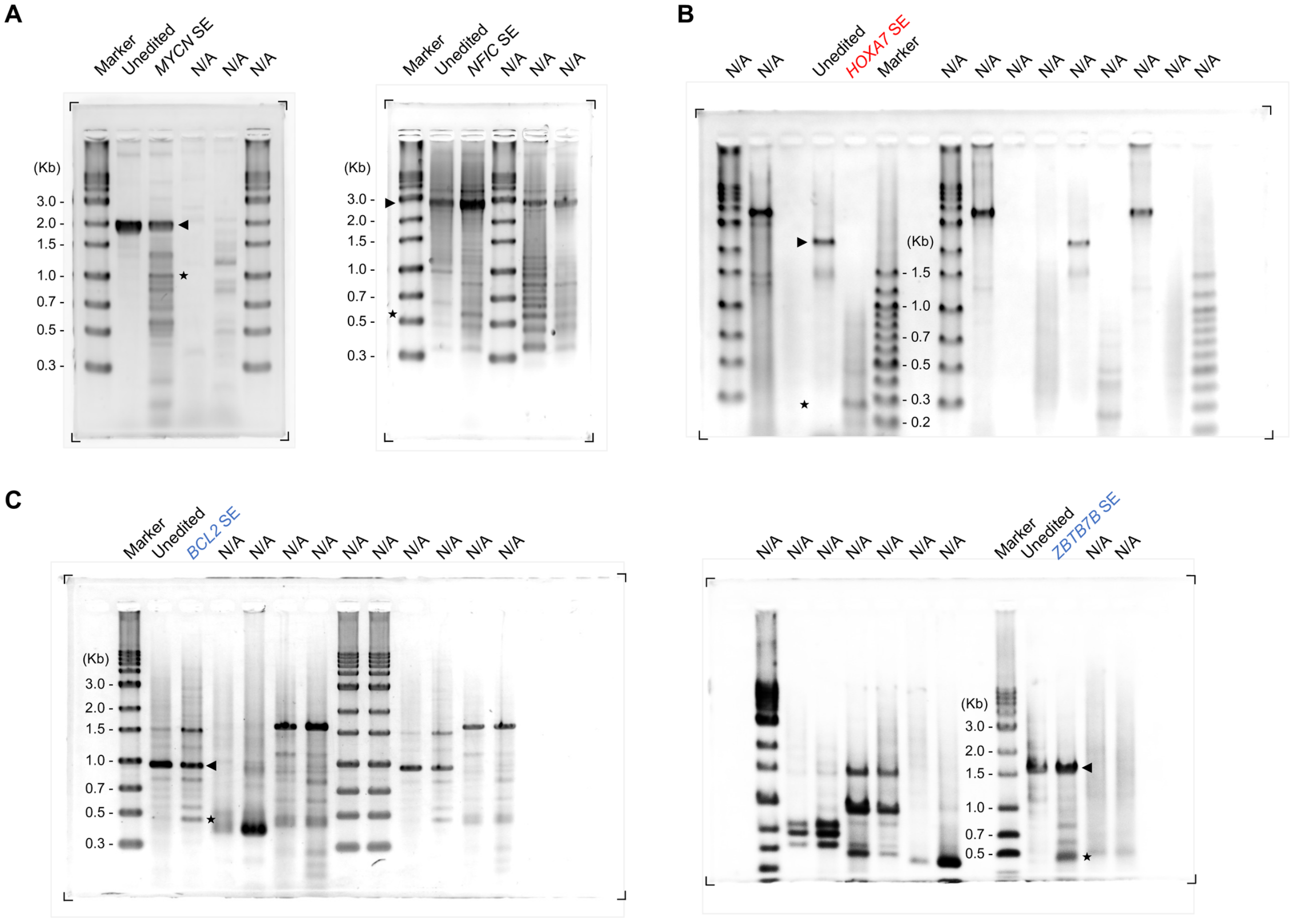

Figure S9. Source data for cropped gel images. A Figure 7B. **B** Figure S7D. **C** Figure S8F. N/A denotes not applicable. Edges of the blots are indicated by black corner brackets.
